# Phage-assisted evolution and protein engineering yield compact, efficient prime editors

**DOI:** 10.1016/j.cell.2023.07.039

**Published:** 2023-08-31

**Authors:** Jordan L. Doman, Smriti Pandey, Monica E. Neugebauer, Meirui An, Jessie R. Davis, Peyton B. Randolph, Amber McElroy, Xin D. Gao, Aditya Raguram, Michelle F. Richter, Kelcee A. Everette, Samagya Banskota, Kathryn Tian, Y. Allen Tao, Jakub Tolar, Mark J. Osborn, David R. Liu

**Affiliations:** 1 Merkin Institute of Transformative Technologies in Healthcare, Broad Institute of MIT and Harvard, Cambridge, MA, USA; 2 Department of Chemistry and Chemical Biology, Harvard University, Cambridge, MA, USA; 3 Howard Hughes Medical Institute, Harvard University, Cambridge, MA, USA; 4 Department of Pediatrics, University of Minnesota Medical School, Minneapolis, MN, USA

**Keywords:** prime editing, genome editing, CRISPR-Cas9, directed evolution, phage-assisted continuous evolution, protein engineering, pegRNAs, guide RNAs

## Abstract

Prime editing enables a wide variety of precise genome edits in living cells. Here we use protein evolution and engineering to generate prime editors with reduced size and improved efficiency. Using phage-assisted evolution, we improved editing efficiencies of compact reverse transcriptases by up to 22-fold and generated prime editors that are 516–810 base pairs smaller than the current-generation editor PEmax. We discovered that different reverse transcriptases specialize in different types of edits and used this insight to generate reverse transcriptases that outperform PEmax and PEmaxΔRNaseH, the truncated editor used in dual-AAV delivery systems. Finally, we generated Cas9 domains that improve prime editing. These resulting editors (PE6a-g) enhance therapeutically relevant editing in patient-derived fibroblasts and primary human T-cells. PE6 variants also enable longer insertions to be installed *in vivo* following dual-AAV delivery, achieving 40% *loxP* insertion in the cortex of the murine brain, a 24-fold improvement compared to previous state-of-the-art prime editors.

## Highlights

• PE-PACE converts compact, low-activity RTs into efficient prime editors
• PegRNA length and secondary structure determine the optimal choice of prime editor
• PE6 RT and Cas9 domains can enhance prime editing efficiencies beyond that of PEmax
• AAV-delivered PE6 editors enable the installation of long, complex edits *in vivo*

## Graphical abstract

**Figure:**
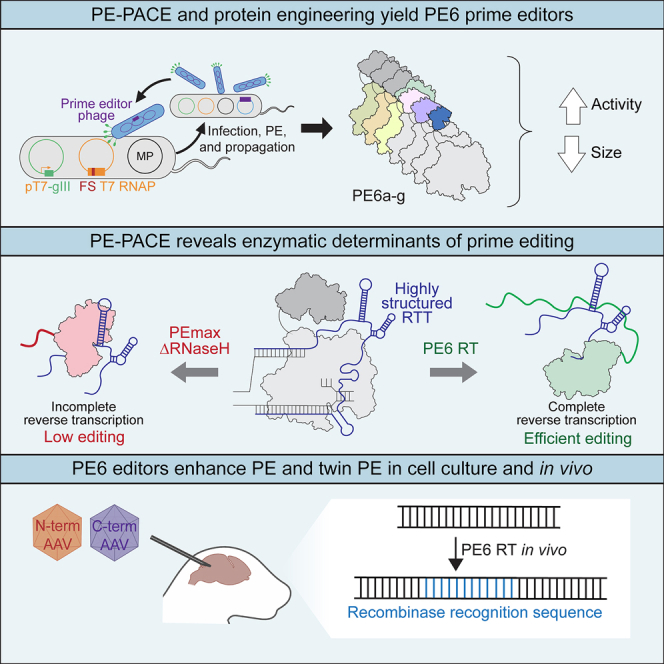

## Teaser

Phage-assisted continuous evolution and protein engineering of prime editors reveals relationships between prime edit type, reverse transcriptase variant, and editing efficiency, enabling the development of PE6 reverse transcriptase and Cas9 variants with reduced size and improved editing efficiency in cell lines and in mice.

## Introduction

**Figure 1:**
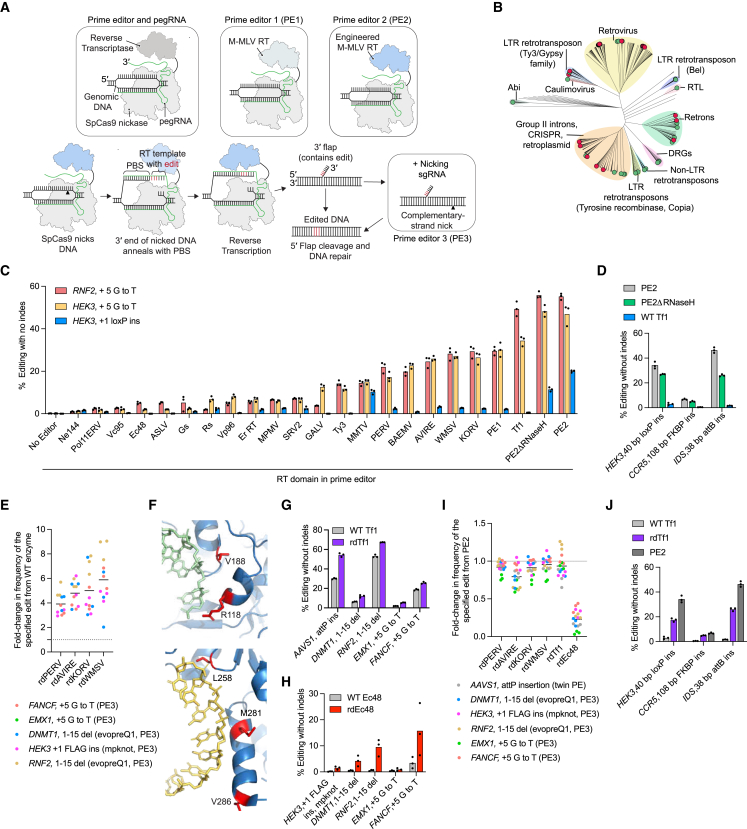
Identification and engineering of reverse transcriptase enzymes into prime editor candidates (A) Overview of PE systems. All use a prime editor protein consisting of SpCas9(H840A) nickase fused to a reverse transcriptase (RT) enzyme. PE1 uses the wild-type RT from the Moloney murine leukemia virus (M-MLV), while the PE2 system uses an engineered pentamutant variant of the M-MLV RT. PE3 uses an additional single guide RNA (sgRNA) to nick the non-edited strand. PBS = primer binding site. RT template = reverse transcriptase template. (B) Phylogenetic classification of RTs tested in this study. Red circles indicate PE-active enzymes. Green circles indicate PE-inactive enzymes. (C) Mammalian activity of 20 different RT enzymes in the prime editing system at endogenous sites in HEK293T cells. (D) Comparison of wild-type Tf1 RT, PE2ΔRNaseH, and PE2 at three longer, complex PE (*HEK3*) or twinPE (*CCR5* and *IDS*) edits in HEK293T cells. (E) Comparison of prime editors containing engineered retroviral RT variants with their wild-type counterparts in HEK293T cells. Horizontal bars show the mean value. (F) Residues mutated to improve editing of the Tf1 RT prime editor correspond to V188, R118, L258, M281 and V286 (red) in Ty3 RT (blue, PDB: 4OL8). V188 and R118 are in close proximity to the RNA (green) substrate and correspond to K118 and S188 in Tf1, respectively. L258, M281 and V286 are near the DNA (yellow) substrate and correspond to I260, S297 and R288 in Tf1, respectively. (G) Rationally designed Tf1 pentamutant variant (rdTf1) shows improvements in editing over its wild-type counterpart in HEK293T cells. All edits are PE edits, except the AAVS1 site, which is twinPE. (H) Rationally designed Ec48 triple mutant variant (rdEc48) shows improvements in editing over its wild-type counterpart for five edits in HEK293T cells. (I) Comparison of prime editors containing engineered RT variants with PE2 in HEK293T cells. All edits use single-flap prime editing, except the *AAVS1* site, which uses twinPE. (J) Comparison of rdTf1 with PE2 and its wild-type counterpart at three longer, complex PE (*HEK3*) or twinPE (*CCR5* and *IDS*) edits in HEK293T cells. Dots indicate individual replicates for n = 3 biological replicates (C–E and G–J). Bars reflect the mean of n = 3 independent replicates (C, D, G, H, and J). See also Figure S1. Throughout all figures (Figures 1, 2, 3, 4, 5, 6, 7, and S1–S7), prime editing efficiencies shown reflect the frequency of the intended prime editing outcome with no indels or other changes at the target site.

Prime editing (PE) can install virtually any substitution, small insertion, or small deletion in the genomes of living cells without requiring double-stranded breaks (DSBs) in DNA or donor DNA templates and thus can correct the vast majority of known pathogenic mutations.^1^ PE requires a prime editing guide RNA (pegRNA) and a prime editor protein, which consists of a programmable nickase and a reverse transcriptase (RT). The first-generation prime editor (PE1) used the wild-type Moloney murine leukemia virus (M-MLV) RT, while subsequent prime editors (PE2–PE5) use an engineered pentamutant M-MLV RT (Figure 1A).^1^^,^^2^ The pegRNA contains a guide RNA scaffold, a spacer that specifies the target site, a primer binding site (PBS) that is complementary to the target DNA, and a reverse transcriptase template (RTT) that encodes the desired edit. The prime editor⋅pegRNA complex pairs with one strand of the target genomic DNA and nicks the opposite strand to generate an exposed 3′ end that binds the PBS of the pegRNA. The RT engages the resulting primer-template complex and initiates reverse transcription of the RTT. The newly synthesized 3′ DNA flap containing the edit is incorporated into the genome, replacing the original DNA sequence and permanently installing the desired edit.^1^ In the PE3 and PE5 systems, an additional single guide RNA (sgRNA) directs the prime editor to nick the non-edited DNA strand and bias cellular mismatch repair to favor installation of the edit (Figure 1A).^1^^,^^2^

Since the development of PE systems, we and others have improved them by engineering the pegRNA,^3^^,^^4^^,^^5^ prime editor architecture,^2^^,^^3^^,^^6^^,^^7^ and cellular DNA repair response to favor desired outcomes.^2^^,^^8^ Twin prime editing (twinPE) and related “dual-flap” methods use two pegRNAs to edit both DNA strands, enabling larger insertions and deletions (>100 base pairs [bp]).^9^^,^^10^^,^^11^^,^^12^^,^^13^^,^^14^^,^^15^ PE and twinPE have been used to install recombinase landing sites, enabling targeted gene-sized (>5,000 bp) insertions and inversions.^9^^,^^16^

Despite these advances, improving the prime editor protein has proven challenging. The M-MLV RT mutations used in PE2–PE5 systems were identified over decades of screening for improved RTs,^17^^,^^18^^,^^19^^,^^20^ followed by additional screening to optimize mammalian PE efficiencies.^1^ These mutations are critical to the efficiency of PE, and few analogous mutations are known for other RTs. Prime editor proteins that use compact RTs could facilitate *in vivo* prime editor delivery, and different RT enzymes may support different editing capabilities. All previously reported prime editors that use RTs other than M-MLV RT, however, have shown substantially lower PE efficiencies than PE2 even after extensive engineering.^3^^,^^16^^,^^21^^,^^22^ Further improvement of the highly engineered M-MLV RT in PE2 has also proven difficult, as all reported variants of this RT have also yielded minimal improvements in mammalian cell PE.^16^^,^^22^^,^^23^ Although we reported that Cas9 mutations known to improve nuclease performance can also increase PE efficiency,^2^ mutants of Cas9 identified specifically to improve PE have not yet been reported.

In this study, we developed a phage-assisted continuous evolution (PACE)^24^ selection for PE and used evolution and protein engineering to generate PE6a–g variants that are more efficient and/or more easily delivered *in vivo* than previous state-of-the-art prime editors. PE6 variants synergize with other recent PE advances^2^^,^^4^ to offer cumulative benefits in a variety of contexts, including in patient-derived fibroblasts and primary human T cells. Dual adeno-associated virus (dual-AAV) delivery of PE6 systems achieved 12- to 183-fold improvements in PE efficiency compared to previous state-of-the-art systems for the installation of 38- to 42-bp edits in the mouse brain, yielding 62% targeted installation of the *loxP* sequence among transduced cells in the mouse cortex.

## Results

### Surveying reverse transcriptase enzymes for prime editing

**Figure S1:**
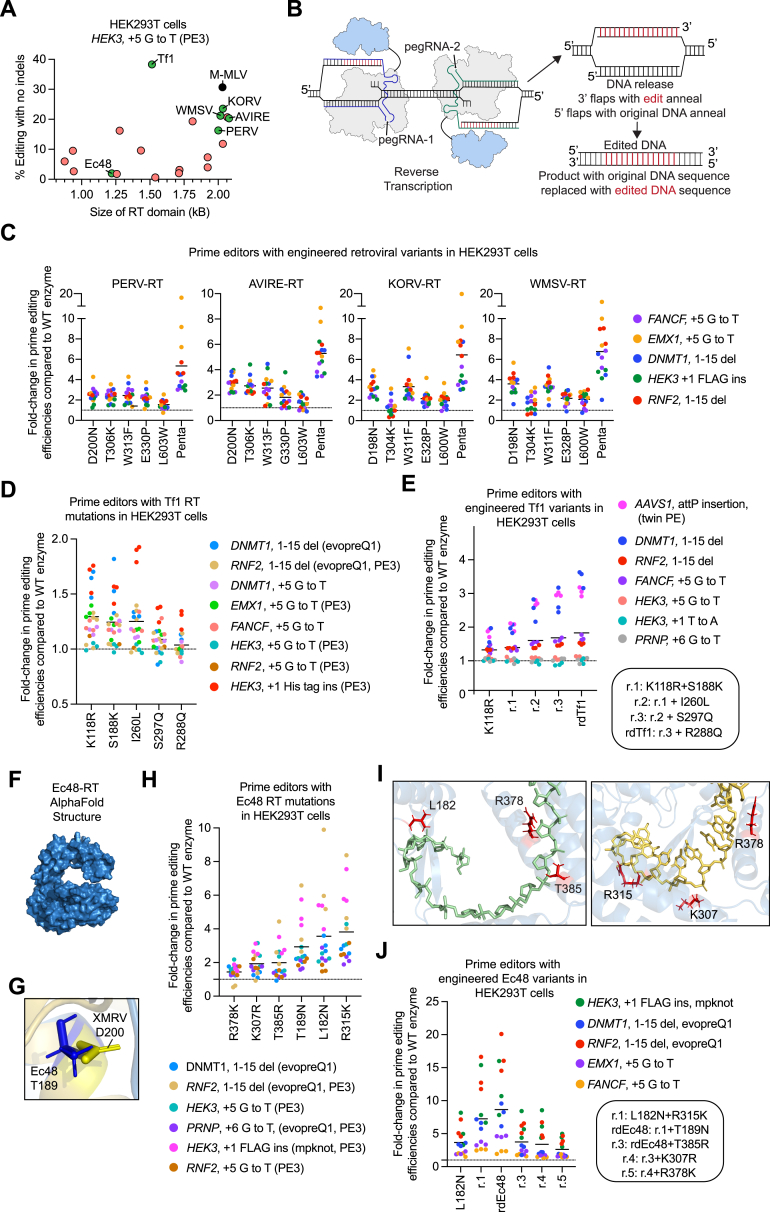
Characterization and engineering of reverse transcriptase enzymes for prime editing, related to Figure 1 (A) Native small RT enzymes demonstrate poor activity in the prime editing system (HEK293T cells, *HEK3* +5 G to T edit). RT enzymes engineered in Figure 1 are highlighted in green, and the wild-type M-MLV RT used in the PE1 system is highlighted in black. All other enzymes are in red. Dots reflect the mean of n = 3 independent replicates. Of these enzymes that can support detectable mammalian PE activity, 11 are closely related to the M-MLV RT and are encoded by retroviruses, two are encoded by LTR retrotransposons, and seven are bacterial RTs from group-II introns, retrons, or CRISPR-Cas associated systems. (B) Overview of twinPE. The prime editor protein (gray and blue) uses two pegRNAs (dark blue and teal) to target opposite strands of DNA. The prime editor generates two 3’ flaps (red) that are complementary to each other. After these newly synthesized 3’ flaps anneal and the original DNA sequence in the 5′ flaps is degraded, the edited sequence in the flaps is permanently installed at the target DNA site. (C) Incorporation of each of the five mutations analogous to those in PE2 (D200N, T306K, W313F, T330P, and L603W) improves the activity of four retroviral RT enzymes in HEK293T cells. PERV = porcine endogenous retrovirus RT, AVIRE = avian reticuloendotheliosis virus RT, KORV = koala retrovirus RT and WMSV = woolly monkey sarcoma virus RT. Combining all five mutations together (Penta) further improves the activity of each enzyme. All values from n = 3 independent replicates are shown. Horizontal bars show the mean value. (D) Structure-guided rational engineering of the Tf1 RT identifies five mutations that improve prime editing in HEK293T cells. The solved structure of the Tf1 RT homolog, Ty3 RT, was used to predict mutations that could increase contacts of the RT with its DNA-RNA substrate (PDB: 4OL8). All values from n = 3 independent replicates are shown. Horizontal bars show the mean value across all sites and replicates. (E) Combining all mutations identified from structure-guided rational engineering improves the activity of the Tf1 RT prime editor in HEK293T cells. The final rationally designed Tf1 variant (rdTf1) is a combination of five mutations: K118R, S188K, I260L, R288Q and S297Q. All values from n = 3 independent replicates are shown. Horizontal bars show the mean value. (F) AlphaFold-predicted structure of the Ec48 RT enzyme. The predicted structure aligns well with the RT from the xenotropic murine leukemia virus-related virus (XMRV, PDB: 4HKQ), a close relative of the M-MLV RT.^70^ (G) Aligning the AlphaFold-predicted structure of the Ec48 RT (blue) with the RT from xenotropic murine leukemia virus-related virus (XMRV, PDB: 4HKQ, yellow), a close relative of the M-MLV RT, suggests that the residue analogous to the D200 residue in M-MLV RT is the T189 residue in Ec48 RT. (H) Structure-guided rational engineering of the Ec48 RT identifies six mutations that improve prime editing in HEK293T cells. An AlphaFold-generated predicted structure of the Ec48 RT was overlayed with the structure of the RT from the xenotropic murine leukemia virus-related virus (XMRV) (PDB: 4HKQ) to perform structure-guided mutagenesis. All values from n = 3 independent replicates are shown. Horizontal bars show the mean value. (I) Positions of residues (red) proximal to the substrate that were mutated to improve the activity of the Ec48 RT prime editor. Residues are mapped onto the predicted AlphaFold structure of the Ec48 RT aligned with the solved substrate of the XMRV RT (PDB: 4HKQ). L182 and T385 are proximal to the DNA substrate (green), R315 and K307 are proximal to the RNA substrate (yellow) and R378 is proximal to both the DNA and RNA rate. (J) Combining the top three mutations identified from structure-guided engineering improves the activity of the Ec48 RT prime editor in HEK293T cells. The final rationally designed Ec48 RT variant (rdEc48) contains three mutations: L182N, T189N and R315K. All values from n = 3 independent replicates are shown. Horizontal bars show the mean value.

Because only a handful of RTs beyond M-MLV RT have been used for PE,^3^^,^^16^^,^^21^^,^^22^ we first surveyed RTs from diverse phylogenetic origins and tested 59 enzymes (Table S1) spanning 14 classes (Figure 1B) as prime editors. We compared these editors to PE1, PE2, and PE2ΔRNaseH (the RNaseH-truncated form of PE2 used for dual-AAV delivery^3^^,^^21^^,^^25^^,^^26^^,^^27^) for three edits in HEK293T cells. Twenty RTs from four different classes showed detectable PE activity, and nine of these RTs are ≥500 bp smaller in gene size than M-MLV RT (Figure 1C). However, all PE-compatible RTs exhibited lower editing efficiencies than PE2, with the smaller RTs showing especially poor activity (Figures 1C and S1A). These results agree with recent reports^3^^,^^16^^,^^21^^,^^22^ that while diverse RTs can support PE, their wild-type forms do not mediate efficient PE in mammalian cells.

The most efficient wild-type RT, *Schizosaccharomyces pombe* Tf1 retrotransposon^28^ RT, approached PE2 efficiencies at substitution edits but struggled to install a 40-bp *loxP* insertion edit (Figure 1C). We noted a similar trend for PE2ΔRNaseH. While the RNaseH domain of MMLV RT is dispensable for PE,^21^^,^^25^^,^^26^ our data suggested that PE2ΔRNaseH might show deficiencies at longer, more challenging edits. Indeed, the Tf1-derived editor and PE2ΔRNaseH performed worse than PE2 at two additional complex edits that use twinPE (Figure S1B). On average, at these three challenging edits, PE2ΔRNaseH yielded 1.4-fold lower PE efficiency than PE2, and wild-type Tf1 performed 15-fold worse than PE2 (Figure 1D).

These initial findings identified three challenges. First, the vast majority of RTs, especially the most compact enzymes, do not support efficient mammalian cell PE for any edit type. Second, even the most active dual-AAV-compatible RTs (∼1.5 kb in gene size) such as the truncated RT in PE2ΔRNaseH showed lower editing efficieny compared to the full-length RT in PE2 when installing long, complex edits. Finally, none of the enzymes we evaluated surpassed the editing efficiency of PE2. We first attempted to addess these problems using protein engineering.

### Rational engineering of reverse transcriptase enzymes

We first engineered retroviral RTs based on our previous engineering of the M-MLV RT to create PE2. The PE2 protein contains five mutations in M-MLV RT (D200N, T306K, W313F, T330P, and L603W) that enhance the enzyme’s *in vitro* substrate binding, processivity, and thermostability.^1^^,^^17^^,^^18^^,^^19^^,^^20^ Installing mutations corresponding to each of these PE2 substitutions into RTs from porcine endogenous retrovirus (PERV), koala retrovirus (KoRV), avian reticuloendotheliosis virus (AVIRE), and woolly monkey sarcoma virus (WMSV) increased PE efficiencies (Figure S1C). Combining all five mutations further improved editing by an average of 5.3-fold to 6.8-fold compared to each enzyme’s wild-type counterpart across five different edits in HEK293T cells (Figures 1E and S1C).

We were also interested in engineering Tf1 RT due to its small size and higher baseline performance compared to other wild-type enzymes. Since increasing the affinity between the RT and its DNA⋅RNA substrate can improve PE efficiency,^1^ we used the structure of a Tf1 homolog, Ty3 RT (Protein DataBank [PDB]: 4OL8), to guide the design of mutations in Tf1 proximal to DNA⋅RNA substrate and tested their ability to support PE in HEK293T cells (Figure 1F). Five of these mutations (K118R, S118K, I260L, S297Q, and R288Q) improved editing efficiency, and combining all five mutations additively improved mammalian editing efficiencies. The final rationally designed Tf1 variant (rdTf1) showed a 1.8-fold average improvement in PE efficiency over wild-type Tf1 in HEK293T cells across seven different edits (Figures 1G, S1D, and S1E).

We also used structure-guided engineering to improve the editing efficiency of the *Escherichia coli* Ec48 retron^29^ RT, which is even smaller than Tf1 RT, but also less active (Figure 1C). Since the structure of a retron RT^30^ had not been reported at the time, we used AlphaFold2^31^ to predict the structure of Ec48 RT (Figure S1F). Incorporation of T189N in Ec48, the mutation predicted by AlphaFold2 to correspond to D200N in PE2, improved PE efficiency by 3-fold on average across six different edits in HEK293T cells (Figures S1G and S1H). Rational engineering using the same structure yielded five additional mutations (K307R, R378K, L182N, T385R, and R378K) that improved PE efficiencies, potentially by improving binding to the DNA or RNA substrates (Figures S1H and S1I). Combining the top-performing mutations yielded rdEc48, which exhibits an 8.6-fold improvement in average PE efficiency over wild-type Ec48 across six edits in HEK293T cells (Figures 1H and S1J).

Despite these substantial improvements, PE efficiencies of all six engineered RT enzymes remained lower than those of PE2 (Figure 1I). The most compact engineered RT (rdEc48) exhibited 8-fold lower average editing efficiencies than PE2 (Figure 1I). Although rdTf1 approached PE2 levels of editing for several edits noted in Figure 1I, it struggled with longer, more complex edits and performed 1.6-fold worse than PE2 at the same three sites tested in Figure 1D (Figure 1J). To overcome these limitations, we turned to laboratory evolution.

### Development and validation of a prime editing PACE selection circuit

**Figure S2:**
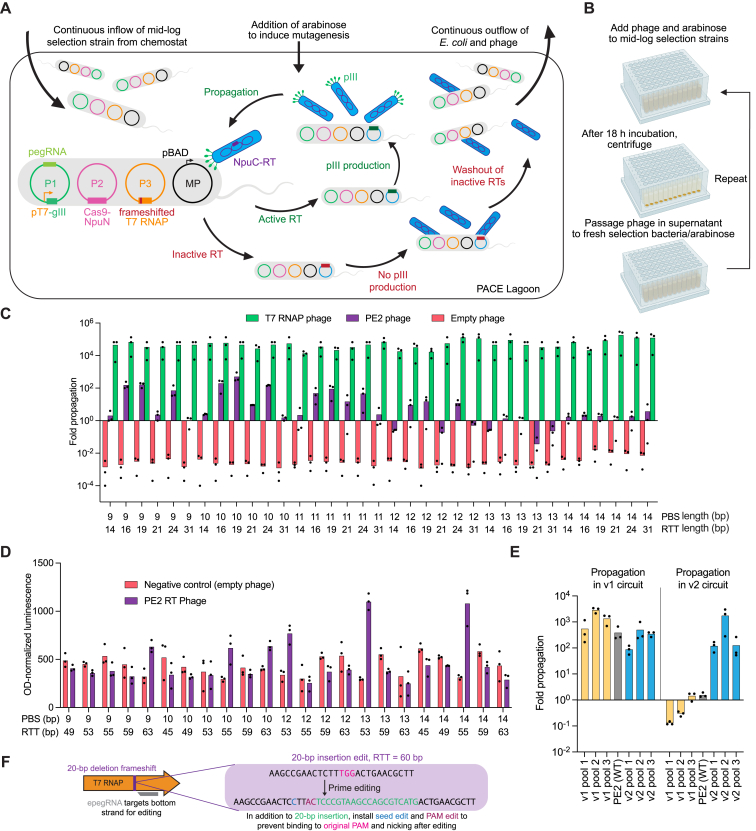
Design and validation of a PE-PACE circuit, related to Figure 2 (A) Summary of phage-assisted continuous evolution (PACE). In both PACE and PANCE, the desired activity of a biomolecule of interest is linked to propagation of a modified M13 bacteriophage. To achieve this linkage, gIII, a gene required for phage propagation, is moved from the phage genome to a plasmid in host *E. coli* cells under the control of a gene circuit, such that gIII expression and phage propagation are only possible if the phage contain gene(s) that encode proteins with the desired activity. Simultaneous expression of mutagenic proteins from the MP6 plasmid mutagenizes the phage, including the gene of interest.^67^ During PACE, continuous dilution of a fixed-volume ‘lagoon’ with fresh host cells selects for rapidly propagating phage encoding molecules that trigger gIII expression (Figure S2A). PANCE uses the same selection strategy, but is implemented using discrete dilution steps every 12–24 h (Figure S2B)^32^: PANCE thus offers higher sensitivity (lower stringency) and greater ease of parallelization than PACE, with the trade-off of slower evolution. Both methods can complete dozens of generations of mutagenesis and selection every 24 h. Host *E. coli* (gray) harboring relevant selection circuit plasmids (green, pink, and orange) and the mutagenesis plasmid (MP, black) continuously flow into a fixed-volume lagoon (left). Addition of arabinose induces expression of mutagenic genes on the MP. Selection phage (blue) harboring an NpuC-RT transgene (purple) infect the *E. coli* and are mutagenized. If a mutagenized RT is inactive (red, bottom/right), then prime editing does not trigger gIII expression and pIII production, and phage are not able to propagate. These phage encoding inactive RTs are washed out of the lagoon by continuous flow. If a mutagenized RT is active (green, center), then prime editing leads to pIII production, and phage encoding that RT can propagate faster than the rate at which they are diluted out of the lagoon. (B) Summary of phage-assisted non-continuous evolution (PANCE). The same principles shown above in Figure S2A are used in PANCE, except periodic discrete dilution steps instead of continuous flow is used to dilute selection cultures. Mid-log phase cultures of selection *E. coli* are infected with phage, and arabinose is added to induce mutagenesis (left). After an overnight incubation, cultures are centrifuged to pellet bacteria and allow isolation of propagating phage from the supernatant (middle). A small volume of supernatant (typically a 1:50 dilution factor) is used to infect a fresh lagoon of mid-log selection strains (right). This process is iterated until phage titers stabilize (i.e., when overnight phage propagation is equal to or greater than the dilution factor). (C) Effect of pegRNA optimization on PE2 phage propagation. Overnight propagation of empty phage (native control, red), PE2 phage (purple), and T7 RNAP phage (positive control, green) in strains harboring pegRNAs of different PBS and RTT lengths. Bars reflect the mean of n = 3 independent replicates. Dots show individual replicate values. This data was used to generate Figure 2C. (D) Luciferase assay to screen pegRNAs for the v2 PE-PACE circuit. Selection strains encoding luxAB transcriptionally coupled to gIII were infected with either empty phage (red) or PE2 phage (purple). 4 h after infection, OD_600_-normalized luminescence was measured as a proxy for circuit activation. Bars reflect the mean of n = 3 independent replicates. Dots show individual replicate values. Strains in which PE2 phage outperformed empty phage were used for v2 evolutions. (E) Overnight propagation of pools of wild-type RT and evolved RT phage on their cognate or noncognate host-cell selection strains. Additional evolved pools of phage are shown here beyond those provided in Figure 2K. Phage were from PANCE on the v1 circuit (yellow bars), from PANCE on the v2 circuit (blue bars), or wild-type-PE2 phage (gray bars). Propagation was then measured in the v1 circuit (left) or the v2 circuit (right). Bars reflect the mean of n = 3 independent replicates. Dots show individual replicate values. (F) Design of v3 circuit and improvements compared to v1 and v2 designs. A long insertion edit (20-bp insertion edit with a 60-bp RTT) was used to select for high-processivity, high-activity prime editors. Unlike v1 and v2 circuits, the v3 pegRNA (gray) targets the noncoding strand of T7 RNAP; this shortens the time between prime editing and wild type T7 RNAP production. In addition to the 20-bp insertion (green) needed to restore the frame of T7 RNAP, the v3 pegRNA also encodes silent PAM edits (maroon) and a seed edit (blue) that prevents subsequent binding and nicking of the edited sequence.

**Figure 2:**
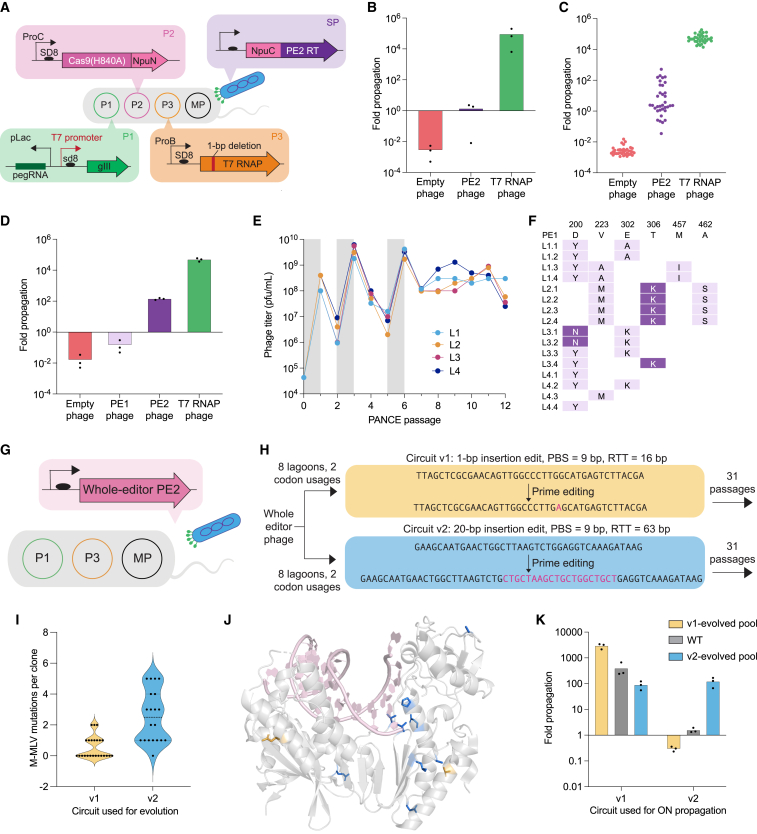
Development and validation of a prime editing PACE selection (A) Schematic of PE-PACE selection circuit. Upon infection of *E. coli* by selection phage (blue), the NpuN intein and NpuC intein (pink) mediate reconstitution of the PE2 prime editor (purple and pink), which engages a pegRNA (dark green) and corrects a frameshift in T7 RNAP (orange) via PE. Functional T7 RNAP then transcribes gIII (light green), which enables SP propagation. (B) Phage replication levels from overnight propagation of empty phage (red), NpuC-PE2-RT phage (purple), and T7-RNAP phage (green) in PE-PACE host cells before pegRNA optimization. (C) Screen of pegRNAs for the v1 PE-PACE circuit. Overnight propagation values of empty phage (red), NpuC-PE2-RT phage (purple), and T7-RNAP phage (green) are shown. Each point reflects the mean value of n = 3 independent biological replicates for a different pegRNA. Individual replicates are shown in Figure S2C. (D) Overnight propagation of empty phage (red), NpuC-PE1-RT phage (light purple), NpuC-PE2-RT phage (dark purple), and T7-RNAP phage (green) in the v1 pegRNA-optimized circuit. (E) PANCE titers for the evolution of NpuC-PE1-RT phage. Gray shading indicates a passage of evolutionary drift, in which phage were supplied gIII in the absence of selection. Titers of four replicate lagoons are shown. (F) Mutation table for NpuC-PE1-RT phage surviving v1 PANCE. Four clones per lagoon (L1-L4, with clones ordered by lagoon) were sequenced. Light purple denotes conserved mutations. Dark purple denotes conserved mutations also present in the previously engineered PE2 RT^1^. (G) Schematic of the PE-PACE selection for evolution of the whole prime editor, including the Cas9 domain. The P1 plasmid (green) and P3 plasmid (orange) are identical to those used in Figure 2A. (H) PANCE experiment to compare the outcome of selection on v1 and v2 selection circuits. Replicate lagoons were evolved on each (v1, yellow and v2, blue) selection circuit. After 31 passages, clones from each selection were sequenced, and the resulting mutations were compared to generate (I-K). (I) Violin plots showing the number of mutations per clone for the M-MLV domain of whole-editor phage evolved with either the v1 (yellow) or v2 (blue) circuit. Data are shown as individual values, with one dot representing one sequenced phage. The mean value is shown as a dotted line. (J) Predicted positions of mutated residues in M-MLV from v1 (yellow) or v2 (blue) PANCE. The structure is from the highly homologous XMRV (PDB: 4HKQ). (K) Overnight propagation of pools of wild-type RT and evolved RT phage on their cognate or noncognate host-cell selection strains. Phage were from PANCE on the v1 circuit (yellow bars), from PANCE on the v2 circuit (blue bars), or wild-type-PE2 phage (gray bars). Propagation was then measured in the v1 circuit (left) or the v2 circuit (right). Bars reflect the mean of n = 3 independent replicates, and dots show individual replicate values (B, D, K). See also Figure S2.

Phage-assisted continuous and non-continuous evolution (PACE and PANCE, respectively)^24^^,^^32^ are methods for highly accelerated laboratory evolution in which the propagation of a modified bacteriophage is linked to the activity of a protein of interest (Figures S2A and S2B). To develop a prime editor PACE (PE-PACE) circuit that links PE activity with phage propagation, we removed the essential phage gene gIII from the phage genome and placed it under the control of a T7 promoter on a plasmid (P1) in host *E. coli*. A second plasmid (P2) contained a defective T7 RNA polymerase (T7 RNAP) gene with a 1-bp deletion frameshift mutation. PE correction of this frameshift enables T7 RNAP production, gIII expression, and phage propagation. In the initial version of our circuit (v1), SpCas9(H840A) nickase was fused to the N-terminal half of the Npu intein (NpuN) and encoded on a separate host plasmid, P3. A C-terminal Npu intein (NpuC) fused to the PE2 RT was encoded on the selection phage, such that intein splicing reconstitutes full-length prime editor after phage infection. Finally, a pegRNA encoding the corrective T7 edit was included on P1. This selection allows the RT, but not the Cas9 nickase domain, to evolve during PACE (Figure 2A).

We evaluated this selection circuit by overnight phage propagation assays. NpuC-PE2-RT phage only propagated 1.4-fold overnight, indicating the need to optimize the circuit (Figure 2B). Because mammalian PE efficiency is heavily influenced by the choice of pegRNA PBS and RTT,^33^ we tested 35 pegRNAs and found that overnight propagation levels of NpuC-PE2-RT phage varied 14,000-fold depending on the pegRNA (Figures 2C and S2C). An optimized pegRNA enabled robust (>100-fold) overnight propagation of NpuC-PE2-RT phage.

To test the dynamic range of the selection, we generated NpuC-PE1-RT phage and evaluated them in our pegRNA-optimized circuit, and we found that NpuC-PE1-RT phage de-enriched 6.7-fold, while NpuC-PE2-RT phage propagated 140-fold (Figure 2D), establishing that the selection can distinguish RT variants based on their PE activity. Finally, to verify that the circuit can enrich mutations that enhance PE, we evolved NpuC-PE1-RT phage in PANCE. Eight overnight PANCE passages yielded six converged mutations (Figures 2E and 2F), including two we previously engineered^1^ in PE2, demonstrating that PANCE can evolve mutations known to enhance mammalian cell PE.

### High-stringency PE-PACE reveals edit-dependent effects on evolved editors

Based on our observation that RTs such as PE2ΔRNaseH and rdTf1 were deficient when using long RTTs (Figures 1C and 1D), we hypothesized that increasing edit size and RTT length would increase the stringency of the PE-PACE circuit. We developed a second circuit (v2, Figure S2D) in which a 20-bp insertion, instead of the 1-bp insertion used in the original v1 circuit, is required to enable phage propagation.

We also speculated that evolving complete PE proteins, rather than only the RT domain, may yield Cas9 mutations that enhance PE outcomes. We therefore removed the P2 plasmid from the host *E. coli* and encoded the entire prime editor protein, including the Cas9 nickase domain, on the phage without the use of a host P2 plasmid or split inteins (Figure 2G).

To study the effects of the target edit on evolutionary outcomes, we designed a comparative PANCE experiment evolving the same whole-editor PE2 phage using the v1 or v2 circuit (Figure 2H). Since different outcomes can emerge even from identical selection conditions,^34^ we performed multiple replicates of each selection. After 31 PANCE passages in six v1 lagoons and five v2 lagoons, we observed that mutations were shared among PANCE replicates for a given edit but differed greatly between lagoons that were required to perform the two different edits (Table S2A; Figures 2H and 2I). Mutations evolved in our v2 circuit were more numerous and also located closer to the polymerase’s active site, whereas residues evolved in the v1 circuit were typically surface exposed (Figures 2I and 2J). These findings demonstrated that the target edit during PE-PACE strongly affects the resulting genotypes, suggesting that the most efficient prime editors may specialize in specific types of edits.

To investigate this possibility, we performed overnight propagation of phage evolved in the 1-bp insertion or 20-bp insertion selection on either the matched or mismatched evolution strain. When phage were evaluated in the strain in which they were evolved, their propagation improved compared to starting whole-editor PE2 phage; however, when evolved phage were evaluated in a strain requiring the other edit, they propagated less well than the parental PE2 phage (Figures 2K and S2E). These data further confirmed that prime editors evolved properties that specialize in their respective edits, and thus different prime editors will likely be best for different types of edits.

We combined the above insights, as well as other recent PE improvements, to design a v3 PE-PACE circuit that used engineered pegRNAs (epegRNAs),^4^ which broadly improve PE by protecting pegRNAs from cellular degradation, to correct a different 20-bp deletion in T7 RNAP (Figure S2F). We used the v1, v2, and v3 PE-PACE circuits to evolve several different RTs below.

### Evolution of compact RTs

**Figure 3:**
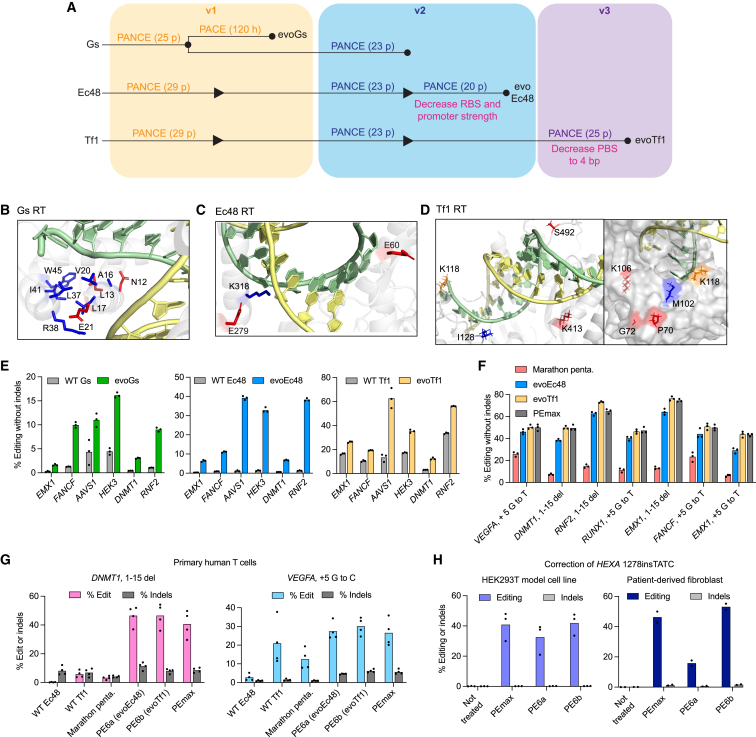
Phage-assisted evolution of compact RTs for prime editing (A) Summary of evolution campaigns for NpuC-Gs RT, NpuC-Ec48 RT, or NpuC-Tf1 RT phage in the v1 (yellow), v2 (blue), and v3 (purple) PE-PACE circuits. Whether an evolution was PANCE or PACE is specified. PANCE passages (p) or hours of PACE (h) are specified in parentheses. Arrowheads indicate increases in selection stringency. Mutants characterized in mammalian cells are denoted with a dot and labeled. Additional increases in stringency are in pink. (B) Position of residues in wild-type Gs RT (PDB: 6AR1) that were mutated during evolution. (C) Predicted positions of residues in Ec48 RT that were mutated during evolution. Residues are mapped onto the AlphaFold-predicted structure of Ec48 RT overlayed with the substrate of the XMRV RT (PDB: 4HKQ). (D) Predicted positions of residues in Tf1 RT that were mutated during evolution. Residues are mapped onto the AlphaFold predicted structure of Tf1 RT overlayed with the substrate of the Ty3 RT (PDB: 4OL8). (E) Prime editing using prime editors containing wild-type (gray) Gs, Ec48, and Tf1 RTs, evolved Gs-RT (evoGs, green), evolved Ec48 RT (evoEc48, blue), and evolved Tf1 RT (evoTf1, yellow) in HEK293T cells (n = 3 independent replicates). (F) Comparison of prime editors in the optimized PEmax architecture containing either engineered pentamutant Marathon RT (Marathon penta, red), evoEc48 (blue), or evoTf1 (yellow) with PEmax (gray) in HEK293T cells (n = 3 independent replicates). (G) Prime editing in primary human T-cells at commonly edited test loci (n = 4 independent replicates). Indel-free editing is shown in blue or pink, and indels are shown in gray. (H) Correction of the *HEXA* 1278insTATC mutation that causes Tay-Sachs disease in a HEK293T cell line model previously engineered to harbor the mutation (left) and in patient-derived fibroblasts (right). n = 3 independent replicates were used for the HEK293T cell line model. n = 2 independent replicates were used for the patient-derived fibroblasts. For B-D, the DNA substrate is green, RNA substrate is yellow, residues mutated following PANCE in the v1 circuit are blue, residues mutated following PANCE in the v2 circuit are red, and residue mutated following PANCE in the v3 circuit is orange. For (E–H), bars show the mean value for the specified number of replicates, and dots show individual replicate values. See also Figure S3.

We first applied PE-PACE to evolve RTs that are substantially smaller than the PE2 RT, including the *Geobacillus stearothermophilus* GsI-IIC intron RT (Gs RT), as well as the Ec48 and Tf1 RTs engineered above (Figure 1). The various evolutionary trajectories pursued are summarized below and in Figure 3A.

**Figure S3:**
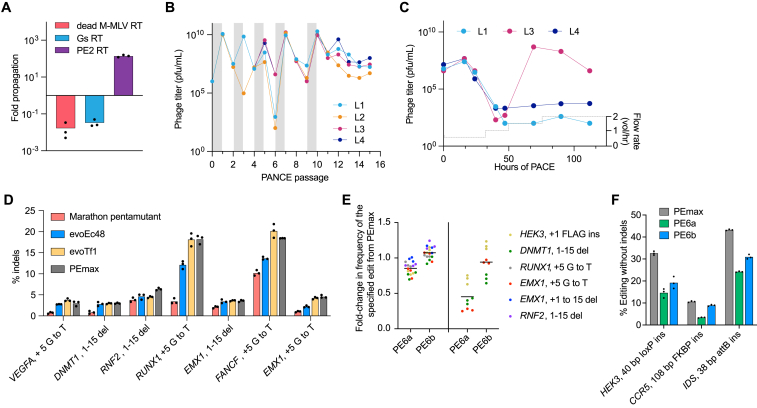
Evolution and characterization of compact RTs for prime editing, related to Figure 3 (A) Overnight propagation of phage encoding dead M-MLV RT (red), Gs (blue), or PE2 (purple) RTs in the NpuC-RT phage architecture in the pegRNA-optimized v1 PE-PACE circuit. Bars reflect the mean of n = 3 independent replicates. Dots show individual replicate values. (B) Phage titers during PANCE of NpuC-Gs-RT phage. Gray shading indicates a passage of evolutionary drift, in which phage were supplied gIII in the absence of selection to allow free mutagenic replication. Titers of four replicate lagoons are shown. (C) PACE of NpuC-Gs-RT phage. The left y axis and pink and blue lines show the SP titer of three different replicate lagoons at various timepoints. The right y axis and dotted gray line show the flow rate in volumes per hour. (D) Indel frequencies for prime editors in the optimized PEmax architecture containing either engineered pentamutant Marathon RT (Marathon penta, red), evoEc48 (blue), or evoTf1 (yellow) with PEmax (gray) in HEK293T cells. Editing frequencies corresponding to this data is in Figure 3F. Bars reflect the mean of three independent replicates. Dots show individual replicate values. (E) Performance of PE6a and PE6b in the presence and absence of epegRNAs in HEK293T cells. All values from n = 3 independent replicates are shown. Horizontal bars show the mean value. (F) Comparison of PE6a, PE6b, and PEmax at three longer, complex edits in HEK293T cells. Bars reflect the mean of n = 3 independent replicates. Dots show individual replicate values.

We began by evolving the weakly active Gs RT (Figure 1C) using 12 passages of PANCE in the v1 circuit, followed by either 100 h in the v1 PACE circuit or 23 passages in the v2 PANCE circuit. Evolution improved phage propagation (Figures S3A–S3C), and sequencing the evolved Gs RT phage showed a high degree of predicted structural convergence (Tables S2B and S2C; PDB: 6AR1)^35^: each clone harbored mutations (N12D, A16E/V, L17P, L37P/R, R38H, I41N/S, and/or W45R) that are predicted to perturb the interaction between two alpha-helices of Gs RT’s N-terminal extension (Figure 3B). One of these helices protrudes into the major groove of the DNA/RNA duplex substrate, suggesting that these mutations may improve substrate binding.

We next evolved the compact Ec48 RT (Figure 1C) using 29 passages of v1 PANCE and 23 passages of v2 PANCE. We increased v2 selection stringency by decreasing the expression of T7 RNAP and evolved the phage for 20 additional passages, yielding high levels of convergence (Tables S2D–S2F). Three mutations (E60K, E279K, and K318E) are predicted to be proximal to the DNA⋅RNA substrate (Figure 3C), suggesting that they also may alter substrate binding.

Finally, we evolved the Tf1 RT using 29 PANCE passages in the v1 circuit, 23 passages in the v2 circuit, and 25 passages in the v3 circuit. In the v3 circuit, we increased selection stringency by decreasing the PBS length from 7 to 4 nucleotides (nt). Several of the resulting converged mutations (K118R, I128V, K413E, and S492N) are proximal to the DNA⋅RNA substrate in the AlphaFold-predicted Tf1 structure, while others (P70T, G72V, M102I, and K106R) may interact with the RTT of the pegRNA (Figure 3D; Tables S2G–S2I). Our previous observation that K118R improves PE efficiency in HEK293T cells (Figure 1E) validates that at least some of the evolved mutations improve mammalian cell editing outcomes. Collectively, these data demonstrate that PE-PANCE enables the rapid, parallel evolution of improved prime editors and is generalizable to diverse RTs.

### Mammalian cell characterization of compact evolved RTs

We evaluated evolved Gs RT, Ec48 RT, and Tf1 RT variants (evo-Gs, evo-Ec48, and evo-Tf1, respectively) as prime editors in HEK293T cells. Across six different edits at endogenous genomic loci using the PE3 system, evolved RTs greatly outperformed their wild-type RT counterparts. We observed a 6.2-fold average improvement for evo-Gs, a 22-fold improvement for evo-Ec48, and a 2.7-fold improvement for evo-Tf1 (Figure 3E).

Among these RTs, evo-Tf1 offered the highest average editing efficiency, and evo-Ec48 was the most compact RT (1.2-kb gene size). We further characterized these two enzymes in the PEmax architecture, which improves codon optimization, linkers, and nuclear localization signals.^2^ We compared these evolved prime editors to PEmax (2.2 kb) and PEmaxΔRNaseH (1.5 kb), as well as the previous state-of-the-art size-minimized (1.2 kb) Marathon pentamutant RT engineered by Joung and coworkers^21^ at six genomic loci using epegRNAs in HEK293T cells.

Evo-Ec48 outperformed the engineered Marathon pentamutant^21^ by 3.7-fold on average and approached PEmax performance levels, averaging 80% of PEmax editing efficiencies across the eight edits tested (Figures 3F and S3D). Since evoEc48 is 810 bp smaller in gene size than the engineered M-MLV RT in PEmax, 270 bp smaller than the ΔRNaseH form of M-MLV, and more efficient than the size-equivalent Marathon pentamutant, we recommend evo-Ec48’s use for PE applications in which the size of the prime editor must be minimized. The use of epegRNAs is important for achieving efficient PE with evo-Ec48 (Figure S3E). We designated the evo-Ec48 RT-derived prime editor as PE6a. Evo-Tf1 on average supported PE levels equal to those of PEmax at the eight edits tested (Figures 3F and S3D). The evo-Tf1 RT-derived prime editor hereafter is designated PE6b. Both PE6a and PE6b are typically less efficient at longer, complex edits (Figure S3F).

To examine PE6a and PE6b variants in a therapeutically relevant cell type, we compared them to their wild-type RT counterparts, the Marathon pentamutant, and PEmax in primary human T cells at two loci following electroporation of the corresponding PE mRNA and pegRNA. For a 15-bp deletion at *DNMT1*, wild-type Ec48 was minimally active (0.22% average editing efficiency), and the Marathon pentamutant yielded 3.3% average editing. The similarly sized PE6a supported 47% average editing, a 211-fold improvement over wild-type Ec48 and a 14-fold improvement over the Marathon pentamutant. PE6a performed as well as or better than PEmax (Figure 3G). Similarly, PE6b offered large improvements over its wild-type RT counterpart, yielding an 8-fold improvement in editing efficiency over PE using wild-type Tf1, comparable to that of PEmax (Figure 3G). We observed similar trends for a substitution edit at *VEGFA*. PE6a and PE6b thus can offer editing efficiencies similar to those of PEmax (Figure 3G) in primary human T cells.

We also evaluated PE6a and PE6b in HEK293T cells harboring the *HEXA* 1278insTATC mutation that causes Tay-Sachs disease.^1^^,^^4^ Treatment of this cell model with PE6a and PE6b and an epegRNA programmed to delete the pathogenic TATC insertion in *HEXA* yielded 33% and 42% correction, respectively, of the pathogenic mutation. These values are similar to the 41% correction generated by PEmax (Figure 3H). We then electroporated either PE6a, PE6b, or PEmax mRNA along with the necessary epegRNA and nicking sgRNA into Tay-Sachs disease patient-derived fibroblasts harboring the 1278insTATC mutation. PE6a, PE6b, and PEmax yielded 16%, 53%, and 46% average *HEXA* correction, respectively—all above the 2% threshold for therapeutic relevance^36^ (Figure 3H).

Overall, these findings establish that size-minimized, non-M-MLV RTs can approach or exceed PEmax’s editing efficiencies while also offering substantially smaller gene sizes (1.2 kb and 1.5 kb for PE6a and PE6b vs. 2.2 kb for PEmax). PE6a and PE6b are the first enzymes in a suite of improved PE6 variants (PE6a-g) developed in this study. To simplify nomenclature, we define PE6 variants as prime editor proteins in the PEmax architecture. When used for PE, the use of a nicking sgRNA is assumed unless stated otherwise, while the use of MLH1dn (which can enhance PE efficiency by inhibiting cellular mismatch repair in the PE4 and PE5 systems)^2^ is not assumed and is specified on a case-by-case basis.

### Evolution and engineering of highly active AAV-compatible RTs

**Figure 4:**
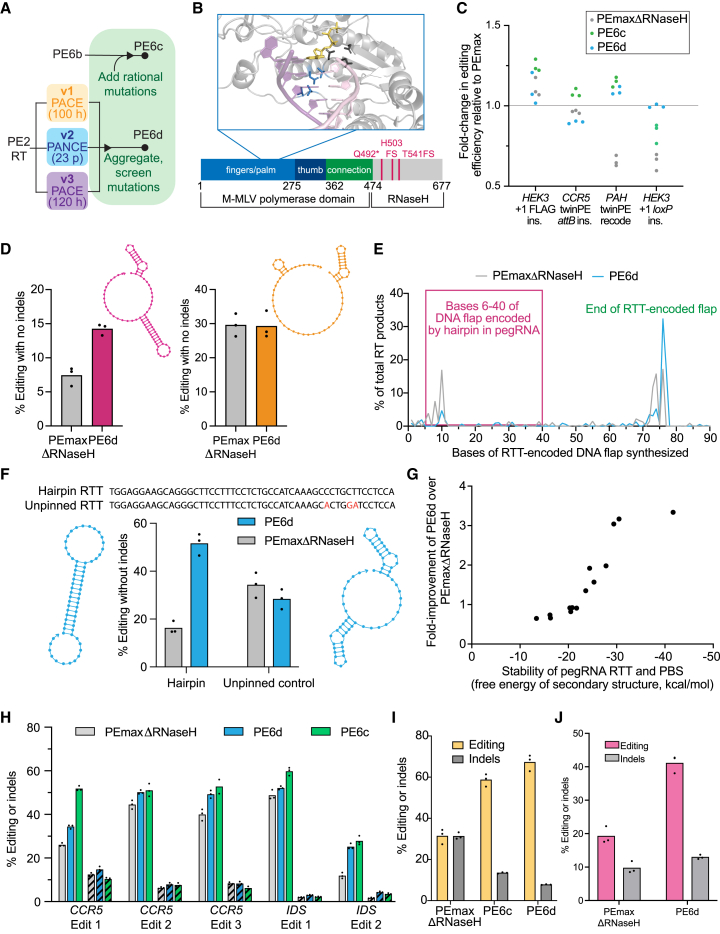
Development of dual-AAV compatible RT variants for installing long, complex edits (A) Summary of evolution and engineering campaigns used to generate PE6c and PE6d. (B) Conserved mutations from M-MLV RT evolution. The structure of XMRV RT (PDB: 4HKQ), which is highly homologous to M-MLV shows PACE-evolved residues (blue) lie close to the enzyme active site (dark gray) and DNA/RNA duplex substrate (pink/purple). An incoming dNTP, modeled by alignment with PDB: 5TXP, is shown in yellow. Below, pink lines indicate locations in the M-MLV RT at which PACE-evolved mutations truncated the protein. (C) Fold-change in editing efficiency relative to PEmax for PEmaxΔRNaseH, PE6c, and PE6d in HEK293T cells. Individual replicates are plotted, with n = 3 biological replicates per edit. (D) Editing efficiencies of PEmaxΔRNaseH and PE6d at the *HEK3* +1 *loxP* insertion edit (pink) and the *HEK3* +1 FLAG insertion edit (orange) in HEK293T cells. The NUPACK-predicted structures of the RTT and PBS extensions for each edit is shown. (E) Results of a TdT assay on the *HEK3* +1 *loxP* insertion edit in HEK293T cells. The y axis indicates the percentage of total RT products of a given length, and the x axis represents the length of the product in base pairs. PEmaxΔRNaseH is shown in gray, and PE6d is shown in blue. The lines are mean values from n = 3 biological replicates. The pink box indicates DNA bases templated by the structured portions of the pegRNA. (F) Editing efficiencies of PEmaxΔRNaseH (gray) and PE6d (blue) at an example engineered hairpin edit and its corresponding unpinned control in HEK293T cells. The sequence of the RTT is shown, with point mutations in the unpinned control shown in red. The NUPACK-predicted structures of the RTT and PBS extensions for each edit is shown. (G) Relationship between pegRNA RTT/PBS secondary structure and PE6d improvements. The y axis reflects the fold-improvement of PE6d over PEmaxΔRNaseH. The x axis is the absolute value of the free energy of pegRNA folding as measured by NUPACK. Each dot represents one edit in HEK293T cells that was calculated from the mean values from n = 3 biological replicates. See Figure S4D for individual editing values and edit identities. (H) Comparison of evolved and engineered RTs to PEmaxΔRNaseH at typical twinPE edits in HEK293T cells. Solid bars indicate editing efficiency. Striped bars indicate indels. (I) TwinPE-mediated insertion of the 38-bp *attB* sequence into the *Rosa26* locus in N2a cells. Indel-free editing is shown in yellow, and indels are shown in gray. (J) PE-mediated insertion of a 42-bp sequence containing *loxP* into the *Dnmt1* locus in N2a cells. Indel-free editing is shown in yellow, and indels are shown in gray. For D, F, and H-J, bars reflect the mean of n = 3 independent replicates. Dots show individual replicate values. See also Figure S4.

Next, we combined PE-PACE with protein engineering to generate prime editors that are the same size as PEmaxΔRNaseH, but better support long, complex edits. To create a highly active Tf1 RT, we combined mutations in the evolved Tf1 RT (PE6b) with rationally designed mutations used in rdTf1. The resulting engineered and evolved Tf1 variant, PE6c, harbors sixteen mutations from evolution and rational engineering (Figure 4A).

**Figure S4:**
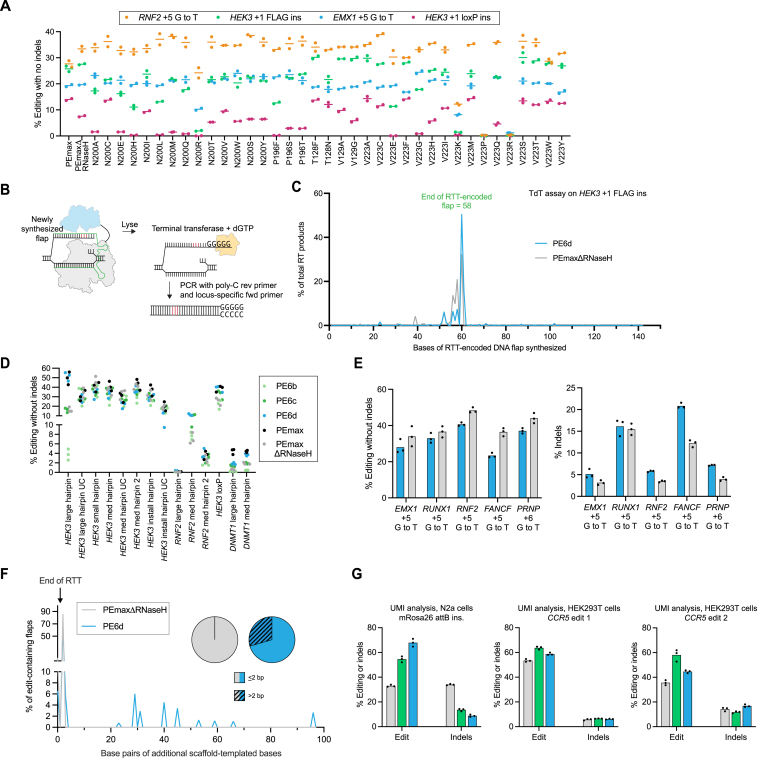
Development and characterization of highly processive, dual AAV-compatible RTs, related to Figure 4 (A) Editing efficiencies of prime editors containing single M-MLV mutants in HEK293T cells. Prime editing efficiencies used are the frequency of the intended prime editing outcome with no indels or other changes at the target site. Lines reflect the mean of n = 2 independent replicates per edit. Dots show individual replicate values. (B) Overview of the terminal deoxynucleotidyl transferase (TdT) assay for directly sequencing newly reverse-transcribed DNA flaps that have not been incorporated into the genome. 24 h after treatment with a prime editor and pegRNA, cells are lysed, and DNA is purified to capture and sequence newly reverse-transcribed DNA before its incorporation into the genome. A terminal transferase enzyme (yellow) adds a polyG sequence to all DNA 3′ ends. PCR amplification for high-throughput DNA sequencing is performed using a locus-specific forward primer and a polyC reverse primer. (C) Results of a TdT assay on the *HEK3* +1 FLAG insertion edit in HEK293T cells. The y axis indicates the percentage of total RT products of a given length, and the x axis represents the length of the product in base pairs. PEmaxΔRNaseH is shown in gray, and PE6d is shown in blue. The lines are mean values from n = 3 biological replicates. (D) Editing efficiencies of PE6b-d, PEmax, and PEmaxΔRNaseH for edits engineered to contain varying levels of secondary structure. “UC” indicates an unpinned control for a corresponding hairpin edit. These values were used to generate the free energy vs. fold improvement plot in Figure 4G. All edits are in HEK293T cells. Individual replicates are shown, with n = 3 replicates per condition. (E) Editing efficiencies (left) and indel rates (right) of PE6d (blue) and PEmaxΔRNaseH (gray) for a series of prime edits that use short unstructured pegRNAs in HEK293T cells. Bars reflect the mean of n = 3 independent replicates. Dots show individual replicate values. (F) Results of a TdT assay on the *RNF2* +5 G to T edit in HEK293T cells. Note that the x axis differs from other TdT plots shown in this study: instead of RTT-templated bases correctly installed, it quantifies the number of sgRNA scaffold-templated bases aberrantly installed (for example, x = 1 indicates the addition of one extra scaffold-templated base). The y axis indicates the percentage of edit-containing flaps that have a given number of scaffold-templated bases. For each prime editor, the line reflects the mean of n = 3 independent replicates. Pie charts indicate the percentages of edit-containing flaps that either have ≤2 bp (solid color) or >2 bp (striped) of scaffold-templated bases. Data shown are the mean of three independent biological replicates. (G) Unique molecular identifier (UMI) analysis of prime editing efficiencies for twinPE edits in N2a cells (left) and HEK293T cells (middle, right). UMI protocol was applied to remove PCR bias, and trends agree with the data shown in Figure 4. Bars reflect the mean of n = 3 independent replicates. Dots show individual replicate values.

To create a highly active, truncated M-MLV RT, we evolved the PE2 RT in the v1, v2, and v3 circuits in parallel and compared mutations emerging from each evolution (Figure 4A). Interestingly, explicit deletion of the RNaseH domain was not necessary, as many evolved M-MLV RT variants contained mutations such as Q492stop that truncated the RT between its polymerase domain and RNaseH domain (Figure 4B).^21^^,^^25^^,^^26^ In addition to these RNaseH-truncating mutations and the five engineered mutations^1^ already present in PE2 compared to wild-type M-MLV RT, over 20 additional mutations emerged (Tables S2J–S2L). One cluster of mutations emerging from the v2 and v3 evolutions was particularly promising (Figure 4B): T128N, V129A/G, P196S/T/F, N200S/Y, and V223A/M/L/E all lie near the polymerase active site. Additionally, we previously installed D200N to create PE2 from the wild-type M-MLV RT,^1^ and V223 is part of the core YXDD motif that has been implicated in the activities of various RTs.^37^ We tested evolved and engineered mutations at these residues, then combined the most promising candidates to generate an RNaseH-truncated evolved and engineered M-MLV variant that we designated PE6d (Figure S4A).

### Dependence of PE6c, PE6d, and PEmaxΔRNaseH performance on RTT secondary structure

We compared PE6c, PE6d, and PEmaxΔRNaseH—three editors small enough to be compatible with dual-AAV delivery^25^^,^^26^ —as well as full-length PEmax, at several longer prime edits and twinPE edits in HEK293T cells. Importantly, PE6c and PE6d recovered PE efficiency for long edits compared to PEmaxΔRNaseH, matching or exceeding PEmax’s editing efficiency for all four tested edits (Figure 4C).

We noted, however, that PEmaxΔRNaseH did not always exhibit deficiencies at long edits compared to PEmax, PE6c, and PE6d, and RTT length alone did not fully account for the performance differences between prime editors. For instance, both the *HEK3* +1 FLAG insertion and the *HEK3* +1 *loxP* insertion pegRNAs require the use of a long RTT (58 bp and 74 bp, respectively) and have identical spacer and PBS sequences, but the relative efficiency of PEmaxΔRNaseH versus PE6d differed substantially between the two edits. While both editors performed comparably at the FLAG insertion, PE6d offered 1.9-fold higher editing efficiency than PEmaxΔRNaseH for the *loxP* insertion (Figure 4D).

To probe this discrepancy, we examined the predicted secondary structure of the two pegRNAs’ 3′ extensions using NUPACK^38^ and found that the FLAG insertion pegRNA 3′ extension is predicted to be largely disordered, whereas the *loxP* insertion 3′ extension contains a strong predicted 13-bp hairpin (Figure 4D). A terminal deoxynucleotidyl transferase (TdT) assay^1^^,^^4^ (Figure S4B) further revealed that for the *loxP* insertion, 30% of products generated by PEmaxΔRNaseH were prematurely truncated at hairpin-templated bases, whereas only 5.8% of products generated by PE6d were prematurely truncated at these positions (Figure 4E). As a result, PE6d produced a larger proportion of full-length DNA flaps that contained the entire RTT-encoded sequence (62% of PE6d RT products versus 34% of PEmaxΔRNaseH RT products [Figure 4E]). In contrast, at the *HEK3* FLAG insertion edit for which the two editors performed similarly, PEmaxΔRNaseH and PE6d both mostly produced full-length flaps (70% and 78% of RT products, respectively [Figure S4C]).

These data suggest a mechanism for the effect of RTT secondary structure on editing efficiency: RNaseH domain truncation, which decreases enzyme processivity,^39^ increases the generation of prematurely terminated, unproductive, RT products when faced with a highly structured RTT substrate. The polymerase domain mutations in PE6d (and certain other variants) enhance RT processivity and can compensate for the lack of the RNaseH domain, supporting full-length product formation even when the pegRNA RTT has substantial secondary structure.

To test this hypothesis, we engineered a series of pegRNAs predicted to contain long, stable hairpins, as well as “unpinned” control pegRNAs in which 2–4 point mutations strongly disrupted pegRNA secondary structure. PE6d outperformed PEmaxΔRNaseH when RTTs contained strong hairpins, yielding a 2.3-fold average improvement in editing efficiency (Figures 4F and S4D). In contrast, the two prime editors performed comparably for the corresponding unpinned control RTTs. These results confirm that secondary structure, rather than RTT length alone, determines the relative efficiencies of PE6d and PEmaxΔRNaseH.

To establish a simple predictive method to identify which compact PE is best for a given edit, we analyzed many prime edits including the hairpin tests above and compared the relationship between the NUPACK-predicted free energy of RTT and PBS folding and the difference in editing efficiency between PE6d and PEmaxΔRNaseH. When the predicted free energy of folding was stronger than −23 kcal/mol, PE6d offered substantial improvements compared to PEmaxΔRNaseH (Figure 4G). This relationship provides a useful guideline for when to use PE6d over PEmaxΔRNaseH.

When the predicted folding free energy of the RTT and PBS was weaker than −23 kcal/mol, PE6d tended to yield lower editing efficiencies and higher indel frequencies than PEmaxΔRNaseH (Figures 4G and S4E). Upon examining the PE6d-mediated indels, we discovered that PE6d catalyzed an increased rate of pegRNA scaffold insertion relative to PEmaxΔRNaseH when a short, unstructured RTT was used (Figure S4F). Scaffold insertion is a byproduct of PE in which reverse transcription of the sgRNA scaffold produces undesired bases at the end of the genomic DNA flap^1^; these extra bases are typically removed by cellular nucleases, but they can impede flap equilibration or generate indels, especially if some scaffold nucleotides share adventitious homology with the target site. PE variants that overcome RTT secondary structure can also increase this type of undesired byproduct, leading to reduced precise editing for short-RTT edits. PE6d is therefore not well suited for most small prime edits. Interestingly, we did not observe general increases in indels (Figures 4H–4J) or scaffold insertion (Figures 4E and S4C) when PE6d was used with a long, structured RTT. We speculate that the RTT itself acts as a barrier to reduce reverse transcription into the sgRNA scaffold. Thus, PE6d and other processive RTs do not generally increase indels at the edit types for which they are most useful; instead, increases in scaffold incorporation occur when the RT is more processive than is required for a specific edit.

This discovery yields key insights into PE. For a given edit, there is an optimal level of RT activity that balances successful generation of RTT-templated bases with minimization of reverse transcription into the sgRNA scaffold. This finding also agrees with our early PACE results and explains why RTs evolved in the v2 selection, which used a long RTT, became less fit in the v1 selection, which uses a short RTT.

We performed similar processivity analyses on Tf1 variants PE6b (which is less processive) and PE6c (which is more processive) and found a similar relationship between these two enzymes (Figure S4D). While generally not as active as PE6d, PE6c outperformed PEmaxΔRNaseH at most highly structured edits (Figure S4D). PE6b has a level of processivity similar to PEmaxΔRNaseH, which makes it a promising candidate for the installation of edits that require a short, unstructured RTT.

PE6c and PE6d should also improve most twinPE efficiencies, which typically use long RTTs. We therefore compared them to PEmaxΔRNaseH at a variety of twinPE edits in HEK293T cells. PE6 variants indeed offered improvements in efficiency relative to PEmaxΔRNaseH, with PE6c yielding a 1.6-fold average improvement across the five sites tested (Figure 4H). To minimize potential PCR bias that can arise during sample preparation for large twinPE edits,^9^ we applied unique molecular identifiers (UMI) to quantify a subset of twinPE edits to confirm this improvement (Figure S4G). Importantly, PE6c and PE6d did not substantially alter the editing:indel ratio for these twinPE edits.

We also examined the ability of PE6 variants to perform longer prime edits in two mouse genomic targets in N2a cells. For the twinPE-mediated insertion of the Bxb1 recombinase *attB* recognition sequence at the murine *Rosa26* safe harbor locus, PEmaxΔRNaseH generated on average 31% installation of the edit but also yielded an equal number of indels. Conversely, PE6c and PE6d both increased editing efficiency and decreased indel rates at this site, with PE6d yielding an 8.6-fold increase in the editing:indel ratio for this edit (Figure 4I). Similarly, we optimized a strategy for the PE-mediated installation of a *loxP* sequence at the murine *Dnmt1* locus. Compared to PEmaxΔRNaseH, PE6d enhanced editing efficiency by 2.1-fold and increased the editing:indel ratio by 1.7-fold (Figure 4J). These data further support that highly processive RTs do not substantially increase indel levels for long, structured RTTs. Overall, these results indicate that among dual-AAV compatible editors, PE6c and PE6d offer substantial improvements over PEmaxΔRNaseH for several types of challenging edits.

### PE6 variants with different processivities offer improvements over PEmax

**Figure 5:**
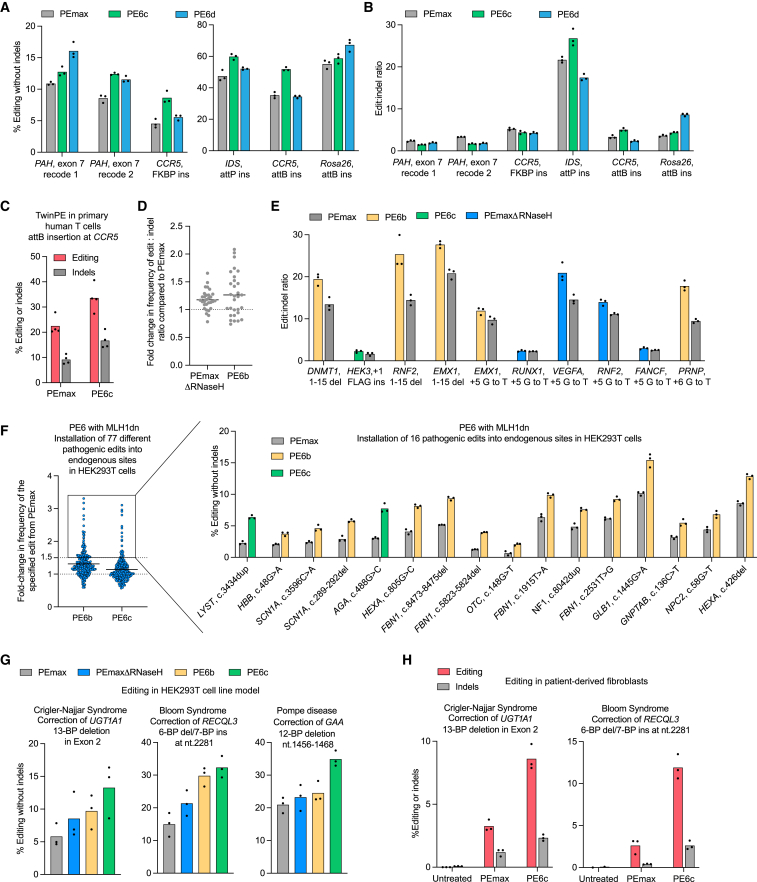
Characterization of PE6 variants compared with PEmax (A) Prime editing efficiencies of PE6c, PE6d, and PEmax at challenging twinPE edits in HEK293T cells. (B) Edit to indel ratios of PE6c, PE6d, and PEmax at sites shown in (A) in HEK293T cells. (C) Twin prime editing in primary human T-cells at the *CCR5* safe harbor locus. Indel-free editing is shown in red, and indels are shown in gray. Bars reflect the mean of n = 4 independent replicates. Dots show individual replicate values. (D) Edit to indel ratios of PE6b and PEmaxΔRNaseH normalized to that of PEmax in HEK293T cells. Individual replicates are plotted, with n = 3 biological replicates per edit. Lines reflect the mean across all edits and replicates. Individual editing efficiencies and indel levels are shown in Figures S5D and S5I. (E) Edit to indel ratios of prime editors at endogenous HEK293T sites. The editor with the highest edit:indel ratio was picked and plotted side-by-side with PEmax for each specific edit. Bars reflect the mean of n = 3 independent replicates. Dots show individual replicate values. Individual editing efficiencies and indel levels are shown in Figures S5D and S5E. (F) Prime editing efficiencies of PE6b and PE6c normalized to the editing efficiency of PEmax at 77 edits that install a pathogenic allele into endogenous sites in HEK293T cells. No nicking gRNA was used and MLH1dn plasmid was simultaneously transfected with prime editor plasmid for all conditions. All values from n = 3 replicates are shown. Lines reflect the mean across all edits and replicates. Prime editing efficiencies for edits where PE6b or PE6c outperformed PEmax by more than 1.5-fold are shown on the right. Bars reflect the mean of n = 3 independent replicates. Dots show individual replicate values. (G) Correction of pathogenic mutations implicated in Crigler-Najjar Syndrome, Bloom Syndrome, and Pompe disease in HEK293T cell models using PEmax, PEmaxΔRNaseH, PE6b, and PE6c. (H) Correction of mutations implicated in Crigler-Najjar Syndrome (*UGT1A1*) and Bloom Syndrome (*RECQL3*) in patient-derived fibroblast using PE6c and PEmax. Bars reflect the mean of n = 3 independent replicates for treated samples and n = 1–3 replicates of an untreated control for editing (red) and indels (gray). Dots show individual replicate values. For A, B, and G, bars reflect the mean of n = 3 independent replicates. Dots show individual replicate values. See also Figure S5.

**Figure S5:**
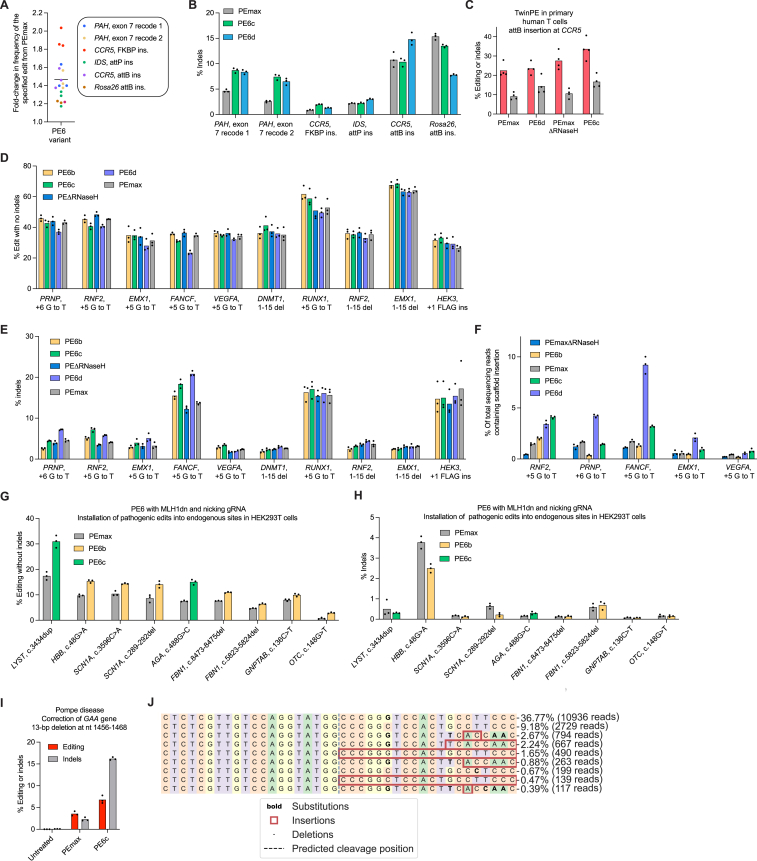
Comparison of PE6 variants with PEmax, related to Figure 5 (A) Prime editing efficiencies of the best performing PE6 variant (either PE6c or PE6d) normalized to the editing efficiency of PEmax at sites tested in Figure 5A. All values from n = 3 independent replicates are shown. Editing was performed in HEK293T cells. The horizontal bar shows the mean value. (B) Indel frequencies of PEmax, PE6c, and PE6d at edits tested in Figure 5A. This data was used for Figure 5B. Bars reflect the mean of three independent replicates. Editing was performed in HEK293T cells. Dots show individual replicate values. (C) Screening PE6 variants for insertion of *attB* into the *CCR5* locus in primary human T cells. Bars reflect the mean of n = 4 independent replicates for editing (red) and indels (gray). Dots show individual replicate values. (D) Absolute prime editing efficiencies of PE6 variants, PEmaxΔRNaseH, and PEmax in HEK293T cells used to plot data for Figures 5D and 5E. Prime editing efficiencies used are the frequency of the intended prime editing outcome with no indels or other changes at the target site. Bars reflect the mean of three independent replicates. Dots show individual replicate values. (E) Indel frequencies of PE6 variants, PEmaxΔRNaseH, and PEmax in HEK293T cells used to plot data for Figures 5D and 5E. Bars reflect the mean of three independent replicates. Dots show individual replicate values. (F) Percentage of sequencing reads containing a pegRNA scaffold insertion after prime editing using PE6 variants, PEmaxΔRNaseH, and PEmax in HEK293T cells. These reads contribute to the total indel frequency. Bars reflect the mean of n = 3 independent replicates. Dots show individual replicate values. (G) Prime editing efficiencies for edits where PE6b or PE6c outperformed PEmax using a nicking gRNA. Bars reflect the mean of n = 3 independent replicates. Dots show individual replicate values. Prime editing efficiencies used are the frequency of the intended prime editing outcome with no indels or other changes at the target site in HEK293T cells. (H) Indel frequencies of PE6 variant and PEmax at sites shown in Figure 5F in HEK293T cells. Bars reflect the mean of n = 3 independent replicates. Dots show individual replicate values. (I) Correction of mutation implicated in Pompe disease in patient-derived fibroblast using PE6c and PEmax. Bars reflect the mean of n = 3 independent replicates for editing (red) and indels (gray). Dots show individual replicate values. (J) Distribution of editing outcomes after correction of the pathogenic mutation implicated in Pompe disease in patient-derived fibroblasts using PE6c. The patient was heterozygous. Indel genotypes are shown. Interestingly, many of the indels detected at this site did not contain the silent PAM edit encoded by the pegRNA, suggesting those indels were not RT-templated products.

Next, we compared PE6 variants with PEmax. Given PE6c and PE6d′s enhanced processivity, we wondered if they might offer improvements over PEmax for longer prime edits. We therefore tested PEmax, PE6c, and PE6d using six 38- to 108-bp insertion twinPE edits at five loci in HEK293T cells and found that PE6 variants improved average editing efficiency by 1.4-fold over PEmax across these edits (Figures 5A and S5A) without altering the precise edit:indel ratio (Figures 5B and S5B).

We also tested PEmax and PE6 variants for *attB* insertion at the *CCR5* safe harbor locus in primary human T cells. PE6c offered a 1.5-fold improvement in editing efficiency relative to PEmax, achieving an average *attB* insertion efficiency of 34% across T cells from four different donors (Figures 5C and S5C). These results confirm that PE6 variants offer substantial improvements for therapeutically relevant PE.

Since we discovered that highly processive RTs can be detrimental for the installation of edits that use short, unstructured RTTs (Figure S4E), we wondered if the same caveat applied to PEmax. Since PE6b and PEmaxΔRNaseH have reduced RT processivity compared to PEmax (as approximated by their lower performance for long edits), they might improve editing:indel ratios compared to PEmax for small, unstructured edits as a result of reduced pegRNA scaffold incorporation. We compared PE6b, PEmaxΔRNaseH, and PEmax for ten edits using short, unstructured RTTs with NUPACK-predicted RTT free energies between 0 and −12 kcal/mol. Both PE6b and PEmaxΔRNaseH indeed offered more favorable edit:indel profiles than PEmax (Figures 5D, S5D, and S5E), and for every edit tested, PEmaxΔRNaseH or a PE6 variant offered a higher editing:indel ratio than PEmax (Figure 5E). Examination of the indels for a subset of edits confirmed that PE6b and PEmaxΔRNaseH incorporated pegRNA scaffold bases less frequently than PEmax (Figure S5F). Collectively, these data indicate that PE6b and PEmaxΔRNaseH are well-suited for edits with unstructured RTTs due to their lower processivity, which reduces scaffold incorporation and improves edit:indel ratios.

### PE6b and PE6c offer improvements over PEmax for therapeutic edits

An expanded set of prime editor options should increase the likelihood of finding a high-efficiency PE approach for specific therapeutic edits. We tested 77 pegRNAs^40^ (Table S3) that install disease-associated mutations into endogenous sites in HEK293T cells and transfected them along with plasmids encoding MLH1dn (but no nicking sgRNA) and PEmax, PE6b, or PE6c. On average, PE6b and PE6c modestly outperformed PEmax (Figure 5F; Table S3), but at 16 of the 77 sites tested, Tf1-dervied editors offered substantial improvements over PEmax (1.5-fold–3.1-fold, Figure 5F). We chose several edits for which PE6b and/or PE6c improved editing efficiencies and added nicking guide RNAs that target the non-edited strand to enhance editing efficiency. For all of these edits, PE6b or PE6c continued to outperform PEmax without increasing indel levels beyond those of PEmax (Figures S5G and S5H).

Similarly, to examine the potential utility of Tf1-derived editors for disease correction, we used Sleeping Beauty transposase^41^ to integrate pathogenic alleles known to cause glycogen storage disease II (Pompe Disease), Bloom Syndrome, or Crigler-Najjar Syndrome into the genomes of HEK293T cells. We evaluated PEmax, PEmaxΔRNaseH, PE6b, and PE6c for their ability to correct each pathogenic mutation. For all three edits, PE6c generated the highest average editing efficiency (13–35%), a 2.1-fold average increase over PEmax across the three model cell lines (Figure 5G). We also tested PEmax and PE6c in fibroblasts derived from Pompe Disease, Bloom Syndrome, and Crigler-Najjar Syndrome patients. PE6c-mediated improvements in indel-free editing efficiencies were more pronounced in these patient-derived fibroblasts, yielding 1.9-fold–4.5-fold improvement over PEmax (Figures 5H, S5I, and S5J). Collectively, these data show that the PE6 RT variants generated in this study can repeatedly outperform PEmax in a variety of disease-relevant contexts and cell types.

### Evolution of Cas9 variants for enhanced prime editing

**Figure 6:**
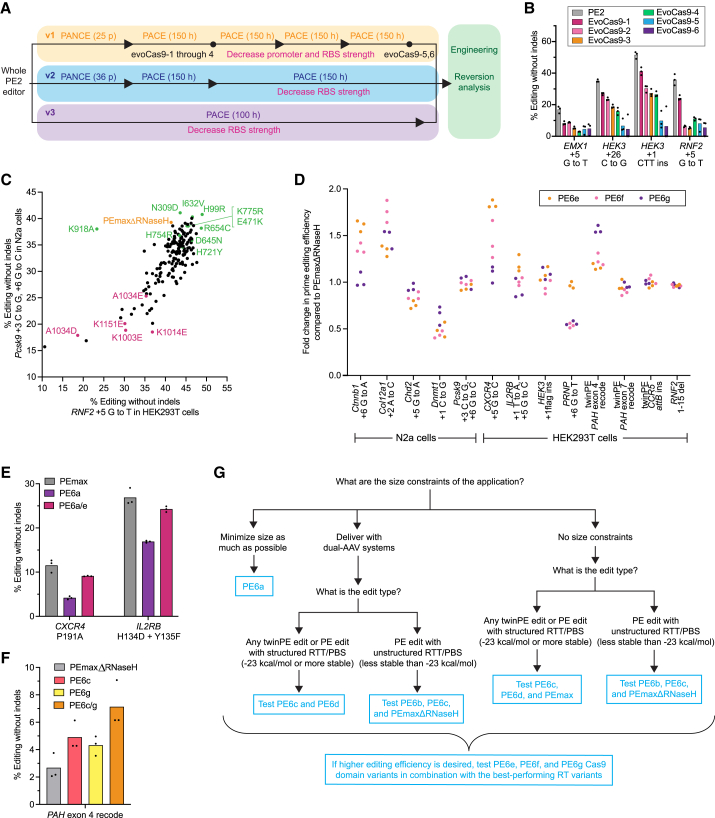
Evolution and engineering of improved Cas9 domains for prime editing, and summary of PE6 recommended use cases (A) Summary of evolution campaigns for whole PE2 phage in the v1 (yellow), v2 (blue), and v3 (purple) circuits. Green shading indicates reversion analysis. PANCE passages (p) or hours of PACE (h) are in parentheses. Arrowheads indicate increases in selection stringency. Mutants characterized in mammalian cells are denoted with a dot and labeled. Additional increases in stringency are in pink. (B) Evaluation of PACE-evolved clones in HEK293T cells. EvoCas9-1 through evoCas9-4 were isolated from low-stringency evolution. EvoCas9-5 and evoCas9-6 were isolated from high-stringency evolution. (C) Assessment of individual Cas9 mutations on prime editing efficiency at two test sites. The y axis shows editing efficiency at the *Pcsk9* +3 C to G / +6 G to C edit in N2a cells. The x axis shows editing efficiency for the *RNF2* +5 G to T edit in HEK293T cells. Mutants incorporated into final Cas9 variants are shown in green. Mutants previously shown to, or structurally predicted to, decrease Cas9 binding are shown in maroon. PEmaxΔRNaseH is shown in orange. (D) Comparison of combined Cas9 mutants to PEmaxΔRNaseH in HEK293T cells and N2a cells. Editing efficiencies of variants are normalized to the editing efficiency generated by PEmaxΔRNaseH. Individual replicates are plotted, with n = 3 biological replicates per edit. (E) Comparison of PEmax, PE6a, and PE6a/e at two sites in HEK293T cells. (F) Comparison of PEmaxΔRNaseH, PE6c, and PE6g in HEK293T cells. (G) Decision tree for selecting a PE6 variant. For secondary structure stability predictions, we recommend the NUPACK prediction tool^38^ with the RTT/PBS sequence as the input. For B, E, and F, bars reflect the mean of n = 3 independent replicates. Dots show individual replicate values. See also Figure S6.

**Figure S6:**
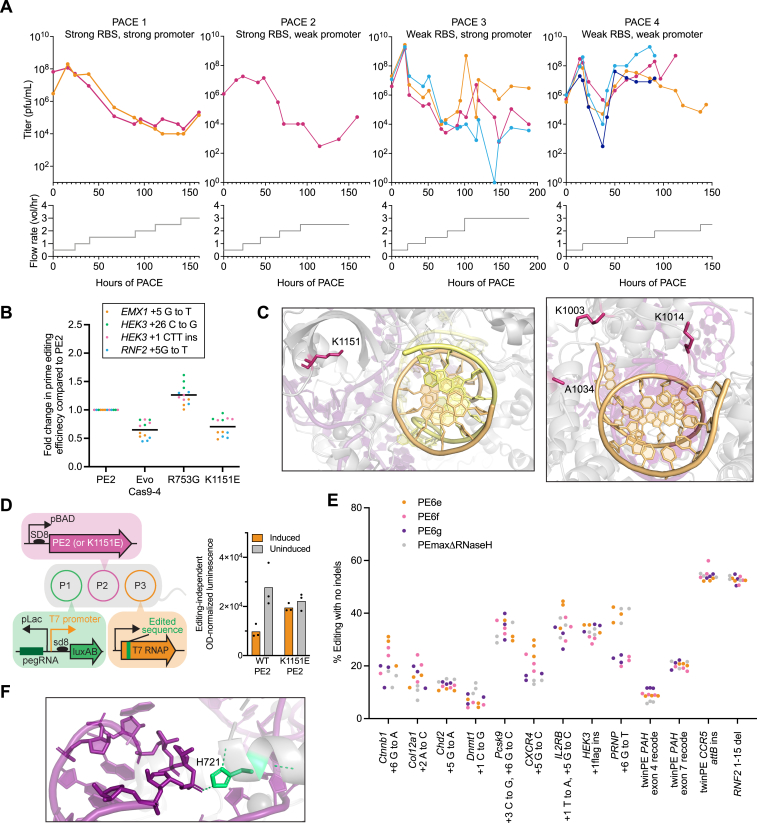
Evolution and engineering of Cas9 mutants for PE, related to Figure 6 (A) Representative PACE campaign for the v1 circuit. Different colored lines represent different replicate lagoons. PACE experiments with less than four lagoons shown experienced cheating (activity-independent phage propagation likely from rare gene III recombination onto the SP) or washout (complete loss of viable phage) for one or more lagoons. Top graphs represent the phage titer over a PACE experiment. Bottom graphs show the flow rate at the corresponding time. (B) Reversion analysis of EvoCas9-4 in HEK293T cells. Editing efficiency was normalized to the values obtained using PE2. Data are shown as individual data points for n = 3 biological replicates and as the grand mean across the four sites tested. (C) Structural analysis of mutations that harm mammalian prime editing activity. (Left) Structure (PDB: 4UN3) of wild-type Sp Cas9 (gray) bound to its guide RNA (purple) and DNA substrate (yellow/orange). Residue K1151 is shown in dark pink. (Right) Structure (PDB: 4OO8) of wild-type Sp Cas9 (gray) bound to its guide RNA (purple) and DNA substrate (orange). Wild-type residues K1003, K1014, and A1034 are shown in dark pink. (D) To test whether mutations that disrupt DNA binding enhanced circuit propagation via mechanisms other than enhancing PE efficiency, we transformed *E. coli* with plasmids encoding a corrected wild-type T7 RNAP, the pegRNA used in the v1 circuit, a gIII-luxAB fusion under the T7 promoter, and either a wild-type or K1151E PE2 mutant under the control of an arabinose-inducible promoter. After induction, OD-normalized luminescence for n = 3 biological replicates were used to measure circuit turn on. This system assessed the effect of each editor on the expression of already-corrected T7 RNAP by luciferase signal. Compared to uninduced bacteria, strains induced to express PE2 exhibited a 2.8-fold lower luciferase signal. Strains induced to express the K1151E mutant, though, showed no reduction in T7 RNAP expression. These findings support a model in which PE-PACE not only selects for PE activity, but also selects for avoidance of impeding the expression of edited T7 RNAP. Bars reflect the mean of n = 3 independent replicates. Dots show individual replicate values. (E) Prime editing efficiencies N2a cells (left, *Ctnnb1* through *Pcks9*) and HEK293T cells (right, *CXCR4* through *RNF2*) used to generate the fold changes reported in Figure 6D. Individual replicates are plotted, with n = 3 biological replicates per edit. (F) Structure (PDB: 4UN3) of Cas9 (gray) bound to its sgRNA (purple). Residue H721, which is mutated to Tyr in evolutions, is shown in green sticks. Dotted lines denote predicted polar contacts between H721 and other atoms. The H721Y mutation is predicted to perturb an interaction between Cas9 and stem loop 2 of the guide RNA scaffold, so its effects may differ depending on the pegRNA used.

During evolutions that used whole-editor phage, the Cas9 domain of the prime editor also acquired dozens of conserved mutations in the v1–v3 circuits (Figures 6A and S6A). Mutations that evolved in the Cas9 domain were dependent on the target used during evolution and were distributed across the entire Cas9 protein, without evident hotspots in any location (Tables S2M and S2N).

However, evolved Cas9 mutants decreased editing efficiencies compared to PE2 in HEK293T cells (Figure 6B). Reversion analysis of evolved Cas9 mutants suggested that a subset of evolved mutations were driving lower mammalian cell editing efficiencies (Figure S6B). To identify beneficial and detrimental mutations, we dissected the effect of 163 individual Cas9 mutations in PEmaxΔRNaseH for two substitution edits in human and mouse cells (Figure 6C; Table S4). Most mutations that strongly decreased editing efficiency at both mammalian targets (K1151E, A1034D, K1003E, and K1014E) are known to decrease the affinity of Cas9 for DNA, or are predicted to do so based on structures of Cas9 complexed with DNA^42^^,^^43^^,^^44^^,^^45^^,^^46^ (Figure S6C; Table S2M). We hypothesized that during PACE, Cas9 binding to a target gene can decrease the expression of that gene through a bacterial CRISPRi mechanism,^47^ so high-affinity binding to the corrected T7 RNAP gene after PE can lower fitness. In mammalian cells, however, requirements for DNA binding are likely more stringent due to lower target site concentration and competing DNA-binding proteins. Therefore, in mammalian cells, PE efficiency may suffer from weaker DNA binding by Cas9. Indeed, we confirmed that disrupting Cas9⋅DNA binding improved PE-PACE circuit activation in a prime editing-independent manner (Figure S6D).

### Engineering Cas9 variants for enhanced prime editing

Having identified and rationalized the enrichment of detrimental Cas9 mutations, we next combined Cas9 mutations beneficial to PE. The single-mutant Cas9 assays identified mutants such as H99R, E471K, I632V, D645N, R654C, H721Y, K775R, and K918A that maintained or modestly increased mammalian PE efficiency (Figure 6C; Table S2N). To create Cas9 variants that can better enhance mammalian PE efficiency, we tested these mutations in combinations to identify the best-performing evolved and engineered Cas9 variants, designated PE6e-g (Figure 6D). We compared these mutants to parental PEmaxΔRNaseH across a wider array of editing conditions and target sites in HEK293T cells and N2a cells (Figures 6D and S6E). At five of the 13 sites tested, PE6e-g variants improved PE efficiency, supporting up to 1.8-fold improvement in average editing efficiency compared to PEmaxΔRNaseH. This result demonstrates that PE6 Cas9 variants are capable of improving mammalian PE efficiency for some edits.

For other edits, however, PE6e-g did not improve or even decreased editing efficiencies compared to PEmaxΔRNaseH (Figures 6D and S6E). In contrast with evolved RT domains, we did not observe a clear relationship between characteristics of the edit and the benefits of different Cas9 mutants. Nevertheless, the location of the PE6 Cas9 mutations suggest potential explanations for their site-specific benefits to PE. The K775R and K918A mutations are located in Cas9’s L1 and L2 linkers, which are involved in R-loop stabilization and also mediate conformational changes in the HNH domain upon DNA binding.^48^^,^^49^ The H721Y mutation appears to impact binding to the sgRNA scaffold (Figure S6F). Therefore, features specific to a target site’s R-loop or pegRNA may account for the observed site-dependent effects. We recommend screening PE6e-g, in addition to the Cas9 domain in PEmax, when optimizing a PE strategy for a site of interest. If only one Cas9 mutant can be tested in addition to the PEmax Cas9, PE6e is the variant most likely to yield improvements (Figure 6D).

### Combining PE6 RT and Cas9 mutants

To maximize PE efficiencies, evolved RT and Cas9 variants can be evaluated separately and then combined. For example, the size-minimized PE6a RT exhibits lower editing efficiencies than PEmax at the *CXCR4* and *IL2RB* loci (Figure 6E), but the evolved PE6e Cas9 improves PE efficiency at those loci (Figure 6D). Combining these two domains (PE6a/e), restores PE efficiency to near-PEmax levels, while maintaining the small size of the PE6a RT (Figure 6E). Additionally, Cas9 and RT domains that both enhance editing efficiency for an edit can be combined: the RT domain of PE6c and the Cas9 domain of PE6g improve twin PE efficiency for the recoding exon 4 of the *PAH* gene. When these domains are combined to generate PE6c/g, the benefits to editing efficiency were additive, yielding a 2.9-fold improvement over PEmaxΔRNaseH (Figure 6F). These results demonstrate that PE6 RT domains and Cas9 domains can be treated modularly to overcome deficits in one domain or yield cumulative improvements from both domains.

### Recommendations and applications of PE6 mutants

The suite of prime editors engineered and evolved in this study (PE6a–g) offer improvements in editor size (PE6a and b), RT activity (PE6c and d), and Cas9-dependent editing efficiency (PE6e–g). From this set of tools, the choice of prime editor variant for a given application is informed by editor size requirements and characteristics of the desired edit (Figure 6G). We recommend first considering size constraints. When editor size must be minimized, PE6a—the smallest prime editor described to date—should be used. If editor size is restricted due to AAV delivery constraints but does not need to be strictly minimized, PEmaxΔRNaseH and PE6b–d should be considered. If the target edit uses a pegRNA with a highly structured 3′ extension (NUPACK-predicted free energy of −23 kcal/mol or more stable for the RTT and PBS) or is a twinPE edit, PE6c and PE6d are likely to be optimal. Conversely, if the target edit utilizes a largely unstructured 3′ extension (NUPACK-predicted free energy of folding less stable than −23 kcal/mol), PEmaxΔRNaseH, PE6b, and PE6c should be examined. Finally, if no size constraints exist, PEmax can also be tested in addition to the four editors just discussed (Figure 6G). If an edit requires an unstructured RTT and scaffold insertion-derived indel levels are high when using PEmax, then PEmaxΔRNaseH and PE6b should be evaluated in order to reduce indels. Conversely, if an edit is a twinPE edit or a challenging PE edit, PE6c and PE6d may offer improvements over PEmax (Figure 6G). Although indel frequencies vary by site and by RT variant, when PE6 editors are applied to their recommended classes of edits, we do not observe any consistent increases in the proportion of indels. Regardless of the RT used, screening Cas9 variants from PE6e-g in combination with the optimized RT can further enhance editing efficiency (Figure 6G).

### PE6 variants enable longer and more complex edits *in vivo* via a dual-AAV delivery system

Following the decision tree in Figure 6G, we used PE6 variants to perform long, complex prime edits *in vivo*. When using efficient dual-AAV systems for *in vivo* prime editing,^3^^,^^25^^,^^26^^,^^27^ editors smaller than PEmax must be used in order for the PE protein, pegRNA, nicking RNA, and their regulatory elements to fit within the packaging capacity of two AAVs (∼5 kb per AAV). Because PE6c and PE6d are the same size as PEmaxΔRNaseH but substantially outperform PEmaxΔRNaseH at highly structured edits in cell culture, we reasoned that these trends may also facilitate edits requiring structured pegRNAs *in vivo* after dual-AAV mediated delivery (see STAR Methods for details).

**Figure 7:**
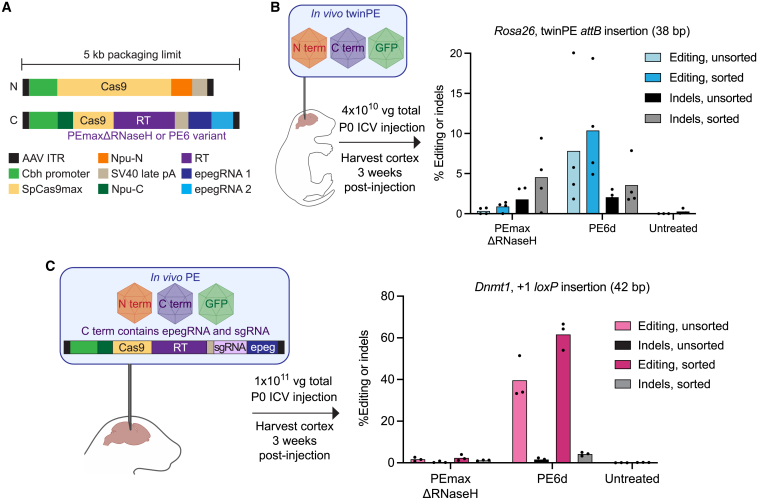
PE6 variants enable longer and more complex prime edits *in vivo* (A) Schematic showing a dual-AAV delivery system for twinPE (v3em twinPE-AAV). In the N-terminal AAV, production of the N-terminal portion of Cas9 (yellow) fused to an N-terminal Npu split intein (orange) is regulated by the Cbh promoter (green) and the SV40 late polyA signal (tan). In the C-terminal AAV, the C-terminal Npu split intein (dark green) is fused to the remainder of the prime editor (Cas9, yellow and RT, purple). The SV40 late polyA signal (tan), two epegRNAs (light and dark blue), AAV ITRs (black) are also shown. (B) Injection route and twinPE editing efficiency of PEmaxΔRNaseH and PE6d viruses in the for the twinPE-mediated insertion of a 38-bp *attB* sequence at murine Rosa26 in the mouse cortex. N- and C- terminal twinPE viruses are administered via ICV injection (4x10^10^ vg total) along with a GFP-KASH virus. Editing efficiencies (light and dark blue) and indel frequencies (black and gray) are shown to the right. Bars reflect the mean of n = 3–4 mice. Dots show individual mice. (C) Injection route and PE editing efficiency of PEmaxΔRNaseH and PE6d viruses for the installation of a 42-bp insertion containing *loxP* at the *Dnmt1* locus in the mouse cortex. (Left) The C-terminal virus is modified to include one epegRNA and one nicking sgRNA to encode a PE edit as opposed to a twinPE edit. (Right) Editing efficiencies (light/dark pink) and indel rates (black/gray). Bars reflect the mean of n = 3 mice. Dots show individual mice. See also Figure S7.

**Figure S7:**
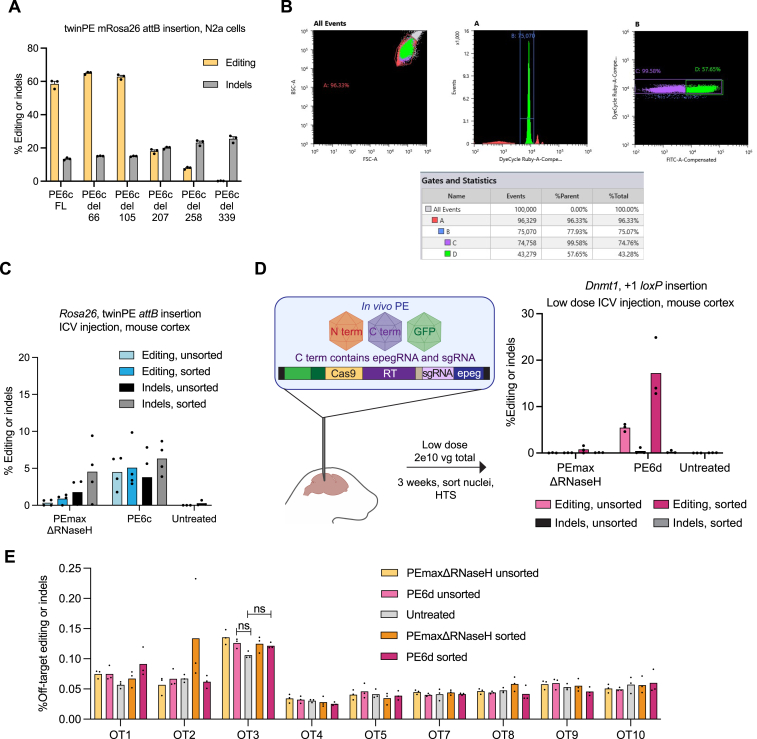
*In vivo* prime editing with PE6c and PE6d delivered via dual AAV, related to Figure 7 (A) Further truncation of the Tf1 RT allowed us to minimize prime editor size an additional 100 bp to facilitate AAV packaging. Editing (yellow) and indels (gray) are shown for the installation of an *attB* sequence at the murine Rosa26 locus in N2a cells using either PE6c or a truncated variant of PE6c. Bars reflect the mean of n = 3 independent replicates. Dots show individual replicate values. The number below each variant indicates the number of DNA bases that have been deleted from the C-terminal end of the Tf1 gene. (B) Representative flow plots for the isolation of unsorted and sorted nuclei from mouse cortices. Left: scatterplot of all events, gate A set to collect nuclei. Middle: selection of single-nuclei droplets in Gate B, Right: FITC signal was used to collect unsorted cells (Gate C) and transduced, GFP-positive cells (Gate D). (C) TwinPE editing efficiency of PEmaxΔRNaseH and PE6c viruses in the mouse cortex. N- and C- terminal twinPE viruses are administered via ICV injection (4x10^10^ vg total) along with a GFP-KASH virus. Editing efficiencies (light and dark blue) and indel (black/gray) rates are shown to the right. Bars reflect the mean of n = 3–4 mice. Dots show individual mice. (D) Injection route and PE editing (*Dnmt1 loxP* insertion) efficiency of PEmaxΔRNaseH and PE6d viruses at a low viral dose (2 x10^10^ vg total) in the mouse cortex. (Left) The C-terminal virus is modified to include one epegRNA and one nicking sgRNA to encode a PE edit as opposed to a twinPE edit. (Right) Editing efficiencies (light/dark pink) and indel rates (black/gray). Bars reflect the mean of n = 3 mice. Dots show individual mice. (E) Off-target editing from AAV-treated and untreated mice. Bars reflect the mean of n = 3 mice. Dots show individual mice. PE6d bulk (light pink) and transduced (dark pink) values were either less than 0.1% on average or were not statistically significant from untreated controls (light gray). For both ns notes, p = 0.08. Analyses were performed with an unpaired t test with Welch correction. The y axis indicates off-target editing and indels summed (see STAR Methods for calculation). OT6 failed to amplify by PCR. All treated samples are from the high AAV dose condition.

We first tested if PE6 variants could enable dual-flap PE *in vivo*, which has not been previously reported. To create a dual-AAV system for twinPE (v3em twinPE-AAV), we began with the architecture described in our recently reported v3em PE-AAV prime editor delivery system^25^ (Figure 7A). In a universal N-terminal AAV, we encoded the majority of the Cas9 protein fused to an N-terminal Npu split intein. In a second C-terminal AAV, we encoded a C-terminal Npu split intein fused to the remainder of the prime editor, using either PEmaxΔRNaseH, PE6c, or PE6d (Figures 7A and S7A). In the C-terminal virus, we included two epegRNAs that are required for twinPE, instead of an epegRNA and a nicking sgRNA (Figure 7A). These epegRNAs encoded the installation of the Bxb1 integrase *attB* substrate sequence at the murine *Rosa26* safe harbor locus. We also included 10^10^ vg of a GFP-KASH AAV to mark nuclei from transduced cells.

We administered a low dose of both twinPE AAVs (4x10^10^ vg total, 2x10^10^ vg per virus) and the GFP AAV (1x10^10^ vg) via neonatal intracerebroventricular (P0 ICV) injections to C57BL/6 mice. Three weeks later, we isolated nuclei from the mice cortices and analyzed bulk (unsorted) or transduced (GFP-positive) nuclei (Figure S7B). Mice treated with PEmaxΔRNaseH AAV showed 0.34% *attB* installation in bulk cortex and 0.89% *attB* installation in transduced cells (Figure 7B). In comparison, PE6c yielded 4.5% and 5.1% insertion of the *attB* sequence in bulk and sorted nuclei, respectively (Figure S7C). PE6d generated 7.8% and 10.4% editing in bulk and sorted cells, respectively (Figure 7B). PE6d thus yielded an average 23-fold improvement in bulk cortex editing and an average 12-fold improvement in editing efficiency in transduced cells relative to PEmaxΔRNaseH. This increase in editing efficiency was not accompanied by an increase in indels relative to PEmaxΔRNaseH (Figure 7B). These data reinforce that PE strategies that were previously inefficient *in vivo* can be achieved using PE6 variants, and establish a method for *in vivo* dual-flap prime editing.

We also tested the ability of PE6 variants to mediate large single-flap insertions *in vivo*. We attempted the installation of a 42-bp *loxP* sequence at the murine *Dnmt1* locus, having observed that PE6d outperformed PEmaxΔRNaseH for this edit in cell culture (Figure 4J). We used the v3em PE-AAV^25^ architecture with either PEmaxΔRNaseH or PE6d. We administered PE-AAVs via P0 ICV injections using a higher dose of 1x10^11^ vg total (5×10^10^ vg per PE virus) or a lower dose of 2×10^10^ vg total (1×10^10^ vg per virus) along with a GFP-KASH AAV transduction marker.

Three weeks after low-dose injection, *loxP* insertion in bulk cortex tissue was virtually undetectable when PEmaxΔRNaseH was used (0.03% average editing [Figure S7D]). Sorting for transduced cells improved PEmaxΔRNaseH-mediated average editing to 0.75%. Importantly, mice injected with a low dose of PE6d showed an average of 5.5% *loxP* insertion in bulk cortex and 17% among transduced cells (Figure S7D) an increase of 183-fold and 23-fold, respectively, compared to PEmaxΔRNaseH. PE6d generated just 0.45% indels and 0.25% indels in bulk and transduced cortex, respectively, leading to an editing:indel ratio of 12:1 in bulk cells and 69:1 among transduced cells (Figure S7D).

Following the higher dose, PEmaxΔRNaseH’s editing efficiency remained inefficient, generating 1.7% and 2.4% *loxP* installation in bulk and transduced cells, respectively (Figure 7C). In contrast, PE6d generated an average of 40% and 62% *loxP* insertion in bulk and transduced cells, respectively, while maintaining low indel levels (1.6% in bulk tissue and 4.2% in transduced cells [Figure 7C]). These results not only represent a large (>23-fold) improvement over PEmaxΔRNaseH in both bulk and transduced cells, but also establish a high editing:indel ratio of 23:1 in bulk cells and 14:1 in transduced cells for PE6d.

To examine whether the more active RT used in these *in vivo* experiments increased off-target PE, we analyzed the top ten CHANGEseq-nominated off-target loci for the *Dnmt1* pegRNA protospacer^26^^,^^50^ for the high-dose treated animals. For both PEmaxΔRNaseH-treated and PE6d-treated animals, we did not detect any off-target modifications (Figure S7E). These results collectively demonstrate that while PEmaxΔRNaseH cannot support the efficient *in vivo* installation of difficult, structured PE or twinPE edits, PE6 variants make these changes possible without generating substantial indels or off-target edits.

## Discussion

In this study, we addressed three key challenges facing PE. First, we developed PE6a and PE6b, which are 516–810 bp smaller in gene size than the M-MLV RT and can support state-of-the-art PE efficiencies. Second, to generate highly active, dual-AAV compatible editors, we used evolution and engineering to produce Tf1-derived PE6c and M-MLV-derived PE6d. Third, we developed multiple strategies for improving editing outcomes over those produced by PEmax. For challenging edits such as those requiring highly structured RTTs, PE6c and PE6d can offer benefits over PEmax; and conversely, for short, unstructured RTTs, indels and scaffold insertion products generated by PEmax can be reduced by using PEmaxΔRNaseH or PE6b. Finally, both Tf1 RT-derived PE6b and PE6c offer different substrate preferences than M-MLV RT-derived editors and can substantially improve editing over PEmax at several therapeutically relevant loci. Evolved and engineered Cas9 domains in PE6e-g can further enhance PE efficiencies at some sites. Recommended use cases for PE6 variants are provided in Figure 6G.

In addition to PE6 editors, this study generated insights that deepen our understanding of PE. By examining differences between PE6 variants and PEmaxΔRNaseH, we discovered that pegRNA extension folding energy is a determinant of PE efficiency. The protospacer-dependent effects from Cas9 mutants that emerged from our selection also raise interesting questions about the target-specific impact of pegRNA binding and R-loop stabilization on PE.

The PE-PACE platform also enables future investigations. The edit-dependent requirements shown here suggest that bespoke prime editor evolution on specific high-impact targets could produce optimal PE systems for those targets. PE-PACE could easily be manipulated for target sequence context-specific selections, which our lab has recently reported for base editing.^51^ PE-PACE could also be used to improve the PE activity of other Cas9 or RT orthologues.^52^ The RTs successfully evolved in this study span four different classes (Group II intron, retron, long terminal repeat retrotransposon, and retrovirus), suggesting that PE-PACE will yield additional advances when applied at scale to the 80,000 reported RT genes in this enzyme superfamily.

Finally, PE6c and PE6d enable longer and more complex insertions to be effectively installed *in vivo* via dual-AAV delivery. They offer an order-of-magnitude improvement compared to a previous state-of-the-art editor, PEmaxΔRNaseH, and support *in vivo* dual-flap PE. Even for non-viral delivery methods in which gene size is not strictly limited, PE6a-d could facilitate critical processes such as the *in vitro* synthesis of editor mRNA or the packaging of editor proteins into liposomes or engineered virus-like particles.^53^

The installation of insertion edits in the CNS is a particularly difficult challenge in genome editing. Homology-dependent methods such as SLENDR and homology-independent methods such as HITI have been used,^54^^,^^55^ but rely on DSBs that can lead to indels. The efficient editing and low indels achieved in this study, combined with the distinct DNA repair pathways required for PE-based approaches relative to other approaches, suggest PE6 variants will be valuable tools for *in vivo* editing. Finally, both *in vivo* edits shown in this study involve the insertion of a recombinase recognition sequence. These results thus lay the foundation for programmable, DSB-free whole gene insertion *in vivo* when paired with a recombinase and donor DNA.

### Limitations of the study

One remaining challenge is how to easily predict which edits will benefit from the use of each PE6 variant. We have addressed this problem for some variants: for dual-AAV compatible prime editors, the degree of predicted pegRNA secondary structure can be used to determine whether PEmaxΔRNaseH or a PE6 variant should be used. For other scenarios, however, guidelines are not as clear. For example, we have demonstrated that Tf1-derived RTs and Cas9 mutants can offer large improvements in editing efficiency compared to PEmax, but these gains are not observed across all target sites and edits. Library-based studies^40^^,^^56^^,^^57^^,^^58^ of RT and Cas9 variants and machine learning models that facilitate *a priori* prediction of the best PE variant for a given application may further advance our understanding of these editors. Finally, while *in vivo* twinPE editing efficiencies remained lower than *in vivo* PE editing efficiencies (here, 10.4% versus 62%), techniques such as increasing dose or extensively optimizing a twinPE dual AAV architecture may be needed to further enhance *in vivo* dual-flap PE efficiencies.

## STAR★Methods

### Key resources table

**Table:** 

REAGENT or RESOURCE	SOURCE	IDENTIFIER
**Bacterial and virus strains**		

One Shot Mach1 T1 Phage-Resistant Chemically Competent E. coli	Thermo Fisher Scientific	Cat#C862003
E. coli S2060	Addgene	#105064

**Chemicals, peptides, and recombinant proteins**		

BsaI-HFv2	New England BioLabs	Cat#R3733S
LguI (SapI)	Thermo Fisher Scientific	Cat#ER1932
T4 DNA Ligase	New England BioLabs	Cat#M0202S
NEBuilder HiFi DNA assembly master mix	New England BioLabs	Cat#E2621S
Dimethyl sulfoxide	Sigma-Aldrich	Cat#D8418-50ML
Poly(ethylene glycol) 3350	Sigma-Aldrich	Cat#P4338-500G
DNaseI (Rnase-free)	New England BioLabs	Cat#M0303
Magnesium chloride solution	Sigma-Aldrich	Cat#M1028-10X1ML
Carbenicillin	Gold Biotechnology	Cat#C-103
Chloramphenicol	Gold Biotechnology	Cat#C-105
Tetracycline	Gold Biotechnology	Cat#T-101
Streptomycin	Gold Biotechnology	Cat#S-150
L-arabinose	Gold Biotechnology	Cat#A-300
Glucose	Sigma-Aldrich	Cat#G7021
Bluo-gal	Gold Biotechnology	Cat#B-673-10
dNTPs	New England BioLabs	Cat#N0447S
Lipofectamine 2000	Thermo Fisher Scientific	Cat#11668019
TrypLE	Thermo Fisher Scientific	Cat#12605010
Proteinase K, recombinant, PCR grade	Thermo Fisher Scientific	Cat#11668019
SDS (10% wt/vol)	Thermo Fisher Scientific	Cat#15553027
DNAdvance Kit	Beckman Coulter	Cat#A48705
AMPure XP	Beckman Coulter	Cat#B23318
CleanCap Reagent AG	TriLink BioTechnologies	Cat#N-7113
N1 -Methylpseudouridine-50 -Triphosphate	TriLink BioTechnologies	Cat#N-1081
LiCl Precipitation Solution (7.5 M)	Thermo Fisher Scientific	Cat#AM9480
DMEM, high glucose, GlutaMAX supplement	Thermo Fisher Scientific	Cat#10566016
Fetal bovine serum	Thermo Fisher Scientific	Cat#16000044
L-Glutamine	Corning	Cat#25-005-Cl
Penicillin-Streptomycin	Thermo Fisher Scientific	Cat#15070063
GlutaMAX supplement	Thermo Fisher Scientific	Cat#35050061
N-acetyl-L-cysteine	Sigma-Aldrich	Cat#A7250-100G
Human AB Serum	Valley Biomedical	Cat#HP1022HI
Recombinant Human IL-2	Peprotech	Cat#200-02
Lymphoprep density gradient medium	STEMCELL Technologies	Cat#07801
Dynabeads Human T-Expander CD3/CD28	Thermo Fisher Scientific	Cat#11141D
X-VIVO™ 15 Serum-free Hematopoietic Cell Medium	Lonza	Cat#BE02-053Q
Dulbecco′s Modifi–d Eagle′s Medium – low glucose	Sigma-Aldrich	Cat#D5546
Eagle’s minimal essential Medium (EMEM)	ATCC	Cat#30-2003
Opti-MEM reduced serum medium	Thermo Fisher Scientific	Cat#31985070
PEG 8000	Sigma-Aldrich	Cat#25322-68-3
PEG-it Virus Precipitation Solution	System Biosciences	Cat#LV825A-1
Salt active nuclease	ArcticZymes	Cat#70910-202
0.9% NaCl	Fresenius Kabi	Cat#918610
BSA	NEB	Cat#B9000S
Vybrant DyeCycle Ruby	Thermo Fisher	Cat#V10309
EZ-PREP buffer	Sigma-Aldrich	#NUC-101

**Critical commercial assays**		

Phusion U Multiplex PCR Master Mix	Thermo Fisher Scientific	Cat#F562L
Q5 High-Fidelity 2 x Master Mix	New England BioLabs	Cat#M0492L
Phusion Green Hot Start II High-Fidelity DNA Polymerase	Thermo Fisher Scientific	Cat#F537L
QIAquick PCR Purification Kit	QIAGEN	Cat#28104
QIAquick Gel Extraction Kit	QIAGEN	Cat#28704
QIAGEN Plasmid Plus Midi Kit	QIAGEN	Cat#12943
QIAprep Spin Miniprep Kit	QIAGEN	Cat#27106
Qiagen Plasmid Plus 96 Miniprep Kit	QIAGEN	Cat#16181
EasySep Human T cell Isolation Kit	STEMCELL Technologies	Cat#17951
Neon™ Transfection System	Thermo Fisher Scientific	Cat#MPK1096
QuickExtract™ DNA Extraction Solution	Lucigen	Cat# QE09050
SE Cell Line 4D-Nucleofector X Kit S	Lonza	Cat#V4XC-1032
Illustra TempliPhi 100 amplification kit	Cytiva	Cat#25640010
NEB T7 HiScribe Kit	New England BioLabs	Cat#E2040S
AAVpro Titration Kit version 2	Clontech/Takara	Cat#6233
Agencourt DNAdvance Kit	Beckman Coulter	Cat#V10309
MiSeq Reagent Kit v2 (300-cycles)	Illumina	Cat#MS-102-2002
MiSeq Reagent Micro Kit v2 (300-cycles)	Illumina	Cat#MS-103-1002

**Deposited data**		

Amplicon sequencing data	This paper	NCBI SRA: BioProject PRJNA916060

**Experimental models: Cell lines**		

Human (female): HEK293T	ATCC	Cat#CRL-3216
Mouse (male): N2a	ATCC	Cat#CCL-131
Human (female): HEK293T clone 17	ATCC	Cat#CRL-11268
Primary human fibroblast (*HEXA*)	Coriell Institute	Cat#GM00221
Primary human fibroblast (*UGT1A1*)	Coriell Institute	Cat# GM09551
Primary human fibroblast (*RECQL3*)	Coriell Institute	Cat# GM02085
Primary human fibroblast (*GAA*)	Coriell Institute	Cat# GM20092

**Experimental models: Organisms/strains**		

Timed pregnant C57BL/6J mice	Charles River Laboratories	Cat#027

**Oligonucleotides**		

*HEXA,* 1278ins TATC pegRNA: mA^∗^mU^∗^mC^∗^rCr UrUrCrCrArGrUrCrArGrGrGrCrCrArUrGrUrUrU rUrArGrArGrCrUrArGrArArArUrArGrCrArArGrUrU rArArArArUrArArGrGrCrUrArGrUrCrCrGrUrUrAr UrCrArArCrUrUrGrArArArArArGrUrGrGrCrArCr CrGrArGrUrCrGrGrUrGrCrGrUrArCrCrUrGrArAr CrCrGrUrArUrArUrCrGrUrArUrGrGrCrCrCrUrGr ArCrUrUrCrUrCrUrCrUrCrCrGrCrGrGrUrUrCr UrArUrCrUrArGrUrUrArCrGrCrGrUrUrAr ArArCrCrArArCrUrA^∗^mG^∗^mA^∗^mA	Integrated DNA Technologies	N/A
*VEGFA*, +2 G to A pegRNA: mG^∗^mA^∗^mU^∗^rGrUr CrUrGrCrArGrGrCrCrArGrArUrGrArGrUrUrUr UrArGrArGrCrUrArGrArArArUrArGrCrArArGr UrUrArArArArUrArArGrGrCrUrArGrUrCrCr GrUrUrArUrCrArArCrUrUrGrArArArArArGrUrGr GrCrArCrCrGrArGrUrCrGrGrUrGrCrArArUrGrUr GrCrCrArUrCrUrGrGrArGrCrArCrUrCrArUrCrUr GrGrCrCrUrGrCrArGrArArCrArArUrCrUrCrCrGr CrGrGrUrUrCrUrArUrCrUrArGrUrUrArCrGrCr GrUrUrArArArCrCrArArCrUrArGrArA^∗^mU^∗^mU^∗^mU	Integrated DNA Technologies	N/A
*DNMT1*, 1–15 deletion pegRNA: mG^∗^mA^∗^mU^∗^rUr CrCrUrGrGrUrGrCrCrArGrArArArCrArGrUrUrUr UrArGrArGrCrUrArGrArArArUrArGrCrArArGrUr UrArArArArUrArArGrGrCrUrArGrUrCrCrGrUrUrAr UrCrArArCrUrUrGrArArArArArGrUrGrGrCr ArCrCrGrArGrUrCrGrGrUrGrCrArGrGrAr GrGrArArGrCrUrGrCrUrArArGrGrArCrUrArGrUrUr CrUrGrCrCrCrUrUrCrUrGrGrCrArCrCrArGrGrAr CrCrUrCrUrUrCrUrCrGrCrGrGrUrUrCrUrArUr CrUrArGrUrUrArCrGrCrGrUrUrArArArCrCrArArCrUr ArGrArA^∗^mU^∗^mU^∗^mU	Integrated DNA Technologies	N/A
*CCR5*, *attB* insertion pegRNA1: mG^∗^mC^∗^mU^∗^rGr UrGrUrUrUrGrCrGrUrCrUrCrUrCrCrCrGrUrUr UrUrArGrArGrCrUrArGrArArArUrArGrCrArArGr UrUrArArArArUrArArGrGrCrUrArGrUrCrCrGr UrUrArUrCrArArCrUrUrGrArArArArArGrUrGr GrCrArCrCrGrArGrUrCrGrGrUrGrCrArCrGrAr CrGrGrArGrArCrCrGrCrCrGrUrCrGrUrCrGr ArCrArArGrCrCrArGrArGrArCrGrC^∗^mA^∗^mA^∗^mA	Integrated DNA Technologies	N/A
*CCR5*, *attB* insertion pegRNA2: mG^∗^mU^∗^mA^∗^rUrGr GrArArArArUrGrArGrArGrCrUrGrCrGrUrUrUrUr ArGrArGrCrUrArGrArArArUrArGrCrArArGrUrUrAr ArArArUrArArGrGrCrUrArGrUrCrCrGrUrUrArUr CrArArCrUrUrGrArArArArArGrUrGrGrCr ArCrCrGrArGrUrCrGrGrUrGrCrArCrGrAr CrGrGrCrGrGrUrCrUrCrCrGrUrCrGrUrCrArGr GrArUrCrArUrGrCrUrCrUrCrArUrU^∗^mU^∗^mU^∗^mC	Integrated DNA Technologies	N/A
*UGT1A1*, correction of 13BP deletion Exon 2 pegRNA: mG^∗^mC^∗^mU^∗^rCrUrArGrGrArArUrUr UrGrArArGrCrCrArGrUrUrUrUrArGrArGrCrUr ArGrArArArUrArGrCrArArGrUrUrArArArArUrAr ArGrGrCrUrArGrUrCrCrGrUrUrArUrCrArArCr UrUrGrArArArArArGrUrGrGrCrArCrCrGrArGrUr CrGrGrUrGrCrArCrArArUrUrCrCrArUrGrUrUr CrUrCrCrArGrArArGrCrArUrUrArArUrGrUrArGr GrCrUrUrCrArArArUrUrCrCrUrArCrGrCrGrGr UrUrCrUrArUrCrUrArGrUrUrArCrGrCrGrUrUrAr ArArCrCrArArCrUrA^∗^mG^∗^mA^∗^mA	Integrated DNA Technologies	N/A
*RECQL3,* correction of 6-BP del/7BP ins at nt.2281 pegRNA: mU^∗^mC^∗^mU^∗^rGrArGrUrCrArGrUr CrUrUrArUrCrArCrCrGrUrUrUrUrArGrArGrCrUrAr GrArArArUrArGrCrArArGrUrUrArArArArUrArAr GrGrCrUrArGrUrCrCrGrUrUrArUrCrArArCrUrUr GrArArArArArGrUrGrGrCrArCrCrGrArGrUrCrGrGr UrGrCrUrCrCrArGrCrUrArCrArUrArUrCrUrGr ArCrArGrGrUrGrArUrArArGrArCrUrGrCrGrCrGr GrUrUrCrUrArUrCrUrArGrUrUrArCrGrCrGrUr UrArArArCrCrArArCrUrA^∗^mG^∗^mA^∗^mA	Integrated DNA Technologies	N/A
*GAA*, correction of 13-bp deletion nt.1456-1468 pegRNA mU^∗^mC^∗^mG^∗^rUrUrGrUrCrCrArGr GrUrArUrGrGrCrCrCrGrUrUrUrUrArGrArGrCr UrArGrArArArUrArGrCrArArGrUrUrArArArArUrAr ArGrGrCrUrArGrUrCrCrGrUrUrArUrCrArArCrUr UrGrArArArArArGrUrGrGrCrArCrCrGrArGrUrCr GrGrUrGrCrUrCrCrUrCrCrCrArCrCrArGrGrCrCr ArGrGrGrCrUrGrUrGrGrGrGrUrUrGrGrUrGrArAr GrUrCrGrGrGrGrArArGrGrCrArGrUrGrGrArGr CrCrGrGrGrCrCrArUrArCrCrU^∗^mG^∗^mG^∗^mA	Integrated DNA Technologies	N/A
*HEXA*, nick sgRNA: mU^∗^mA^∗^mC^∗^rCrUrGrAr ArCrCrGrUrArUrArUrCrGrUrAGrUrUrUrUrArGrAr GrCrUrArGrArArArUrArGrCrArArGrUrUrArArArAr UrArArGrGrCrUrArGrUrCrCrGrUrUrArUrCr ArArCrUrUrGrArArArArArGrUrGrGrCrArCrCrGrArGr UrCrGrGrUr GrCrUmU^∗^mU^∗^mU	Synthego Corporation	N/A
*VEGFA,* nick sgRNA mG^∗^mA^∗^mG^∗^rCrCrCrAr GrGrGrCrUrGrGrGrCrArCrArGGrUrUrUrUr ArGrArGrCrUrArGrArArArUrArGrCrArArGrUr UrArArArArUrArArGrGrCrUrArGrUrCrCrGrUr UrArUrCrArArCrUrUrGrArArArArArGrUrGrGr CrArCrCrGrArGrUrCrGrGrUr GrCrUmU^∗^mU^∗^mU	Synthego Corporation	N/A
*DNMT1*, nick sgRNA: mC^∗^mC^∗^mC^∗^rUrUrCrArGr CrUrArArArArUrArArArGrGGrUrUrUrUrArGrAr GrCrUrArGrArArArUrArGrCrArArGrUrUrArArArAr UrArArGrGrCrUrArGrUrCrCrGrUrUrArUrCrArAr CrUrUrGrArArArArArGrUrGrGrCrArCrCrGrAr GrUrCrGrGrUr GrCrUmU^∗^mU^∗^mU	Synthego Corporation	N/A
*UGT1A1*, nick sgRNA: mA^∗^mU^∗^mU^∗^rGrCrCrAr UrArGrCrUrUrUrCrUrUrCrUrCrGrUrUrUrUrAr GrArGrCrUrArGrArArArUrArGrCrArArGrUrUrAr ArArArUrArArGrGrCrUrArGrUrCrCrGrUrUrAr UrCrArArCrUrUrGrArArArArArGrUrGrGrCrArCrCrGr ArGrUrCrGrGrUrGrCrUmU^∗^mU^∗^mU	Synthego Corporation	N/A
*RECQL3*, nick sgRNA mA^∗^mU^∗^mU^∗^rCrCrArGr CrUrArCrArUrArUrCrUrGrArCrGrUrUrUrUrArGr ArGrCrUrArGrArArArUrArGrCrArArGrUrUrArAr ArArUrArArGrGrCrUrArGrUrCrCrGrUrUrArUrCr ArArCrUrUrGrArArArArArGrUrGrGrCrAr CrCrGrArGrUrCrGrGrUrGrCrUmU^∗^mU^∗^mU	Synthego Corporation	N/A
*GAA*, nick sgRNA mA^∗^mG^∗^mC^∗^rCrArCrCrArUrGrUr CrCrUrCrCrCrArCrCrGrUrUrUrUrArGrArGrCrUr ArGrArArArUrArGrCrArArGrUrUrArArArArUrAr ArGrGrCrUrArGrUrCrCrGrUrUrArUrCrArArCrUr UrGrArArArArArGrUrGrGrCrArCrCrGrArGr UrCrGrGrUrGrCrUmU^∗^mU^∗^mU	Synthego Corporation	N/A

**Recombinant DNA**		

Mutagenesis plasmid MP6	Addgene	#69669
pJC175e	Addgene	#79219
pBT114-splitC	Addgene	#138523
pBT29-splitD	Addgene	#138521
pCMV-PE2	Addgene	#132775
pCMV-PEmax	Addgene	#174820
pT7-PEmax	Addgene	#178113
pEF1a-MLH1dn	Addgene	#174824
pU6-tevopreq1-GG-acceptor	Addgene	#174038
pU6-pegRNA-GG-acceptor	Addgene	#132777
pCMV-PE6a	This paper	N/A
pCMV-PE6b	This paper	N/A
pCMV-PE6c	This paper	N/A
pCMV-PE6d	This paper	N/A
pCMV-PE6e	This paper	N/A
pCMV-PE6f	This paper	N/A
pCMV-PE6g	This paper	N/A
AAV-PE6c-Rosa26-twinPE	This paper	N/A
AAV-PE6d-Rosa26-twinPE	This paper	N/A
AAV-PEmaxdeltaRNaseH-Rosa26-twinPE	This paper	N/A
AAV-PE6d-Dnmt1-loxP	This paper	N/A
AAV-PEmaxdeltaRNaseH-Dnmt1-loxP	This paper	N/A

**Software and algorithms**		

CRISPResso2	Clement et al., 2019^59^	https://github.com/pinellolab/CRISPResso2
Prism	GraphPad	https://www.graphpad.com/
Geneious Prime	Dotmatics	https://www.geneious.com/prime/
AmpUMI	Clement et al., 2018^60^	http://github.com/pinellolab/AmpUMI.
Python 3	Python	https://www.python.org/downloads/
Mutato	Mok et al., 2022^61^	https://hub.docker.com/r/araguram/mutato/
Scaffold insertion analysis	Anzalone et al., 2019^1^	Note S1
TDT analysis	This paper	Note S2

### Resource availability

#### Lead contact

Please direct requests for resources and reagents to lead contact: David R. Liu (D.R.L. drliu@fas.harvard.edu).

#### Materials availability

Plasmids generated in this study are available from Addgene. Additional details are provided in the key resources table.

### Experimental model and subject details

#### Mammalian cell culture conditions

HEK293T (American Type Culture Collection (ATCC), Cat# CRL-3216), Neuro-2a (N2a from ATCC, Cat# CCL-131) and Huh7 (a gift from Erik Sontheimer’s group, originated from ATCC) cells were cultured in Dulbecco’s Modified Eagle Medium (DMEM) plus GlutaMAX (Thermo Fisher Scientific) supplemented with 10% (v/v) fetal bovine serum (FBS) (Thermo Fisher Scientific). Primary Tay Sachs disease patient fibroblast cells were purchased from Coriell Institute (Cat. ID GM00221) and cultured in low-glucose DMEM (Sigma Aldrich) supplemented with 10% (v/v) FBS and 2mM GlutaMAX Supplement (Thermo Fisher Scientific). All cell lines were incubated, maintained, and cultured at 37^°^ C with 5% CO_2_. Cell lines were authenticated by their respective suppliers and tested negative for mycoplasma.

#### Generation of HEK293T models of Tay-Sachs disease

HEK293T cells homozygous for the *HEXA*1278TATCins mutation were previously reported.^1^ HEK293T cells were seeded in a 48-well plate and transfected with 250 ng of a pegRNA plasmid, 83 ng of a nicking sgRNA plasmid, and 750 ng of a PE2-P2A-GFP plasmid programmed to install the *HEXA*1278TATCins mutation. 3 days after transfection, GFP-positive cells were flow sorted using an LE-MA900 cell sorter (Sony) into a 96-well flat bottom culture well plate. Cells were cultured for 10 days and then analyzed for *HEXA*1278TATCins mutation installation. Two different clonal, homozygous (100% installation of *HEXA*1278TATCins) cell lines were used for experiments.

#### Generation of HEK293T model cell lines for Bloom Syndrome, Crigler-Najjar disease, and Pompe Disease

Pathogenic gene fragments were generated by examining disease alleles from patient-derived fibroblasts in the Coriell Institute database. These gene fragments (300 bp total, flanking the pathogenic mutation) were then ordered as eBlocks (Integrated DNA technologies). These fragments were then cloned into a Sleeping Beauty transposon vector, downstream of a blasticidin resistance gene expression cassette. (The target pathogenic gene itself was not expressed.) 3.2E5 low-passage HEK293T cells were plated in a 6-well dish and transfected with 50 ng of disease allele transposon, 25 ng of transposase, and 725 ng of PUC19 in a total volume of 250 μL using 20 μL lipofectamine 2000 (Thermo Fisher). 48 h after transfection, cells were trypsinized, resuspended in 2 mL of media, and 60 μL of the resuspended cells were plated in a fresh 6-well plate well with media containing 10 μg/mL blasticidin. Cells were passaged until a no-transposase negative control had completely died. The heterogeneous pool of cells was then used for transfection with editors to target the disease allele for correction. In the downstream HTS sample preparation, primers specific for the transposon backbone were used to selectively amplify the knocked-in pathogenic allele, as opposed to the wild-type endogenous allele.

#### Isolation and culture of primary human T cells

Memorial Blood Center (St. Paul, MN) buffy coats were obtained followed by peripheral blood mononuclear cells (PBMC) isolation with Lymphoprep and SepMate tubes (STEMCELL Technologies). CD4^+^ T-cells were purified from PBMCs using the EasySep Human CD4 + T cell Isolation Kit (STEMCELL Technologies). T-cells were cultured in X-VIVO TM 15 Serum-free Hematopoietic Cell Medium (Lonza, Basel, Switzerland) supplemented with: 300 IU/mL IL-2 (PeproTech), GlutaMAX (Gibco), N-acetyl-cysteine (Sigma Aldrich), 5% AB human serum (Valley Biomedical), 50 U/mL penicillin and 50 μg/mL streptomycin (Gibco).

### Method details

#### General methods and molecular cloning

The following working concentrations were used for antibiotics (Gold Biotechnology): carbenicillin 50 μg/mL, chloramphenicol 25 μg/mL, kanamycin 50 μg/mL, tetracycline 10 μg/mL, streptomycin 25 μg/mL. For all cloning experiments, Nuclease-free water (Qiagen) was used, gene blocks were ordered from Integrated DNA Technologies (IDT) and primers were ordered from either IDT or Eton Biosciences. All synthetic genes were codon-optimized for human cell expression using GenScript’s algorithm and obtained as gene blocks from either GenScript or IDT. All plasmid construction was done using Gibson assembly. Briefly, for most Gibson cloning, unless otherwise noted, PCR was done using either Phusion U Green Hot Start II DNA polymerase (Thermo Fisher Scientific) or Phusion Green Hot Start II High-Fidelity DNA polymerase (Thermo Fisher Scientific). The resulting PCR products were purified using QIAquick PCR purification Kit (Qiagen) and fragments were assembled using NEBuilder HiFi DNA assembly master mix (New England BioLabs) according to the manufacturer’s protocol. Plasmids for mammalian expression of prime editors were cloned into the pCMV-PE2 vector backbone (Addgene #132775) and plasmids used for the *in vitro* transcription of different prime editor mRNA were cloned into the pT7-PEmax (Addgene #178113) vector backbone.

Plasmids for the mammalian expression of pegRNAs, sgRNA, and epegRNAs were cloned as previously described.^33^ Briefly, vector backbone expressing a guide RNA under the human U6 promoter was digested using BsaI-HFv2 (New England BioLabs) according to the manufacturer’s protocol. The digested fragment was purified by gel electrophoresis with a 1% agarose gel using QIAquick Gel Extraction Kit (QIAGEN). The BsaI-digested vector backbone was then assembled with eblocks ordered from IDT using NEBuilder HiFi DNA assembly master mix (New England BioLabs) according to the manufacturer’s protocol. Vector backbone pU6-pegRNA-GG-acceptor (Addgene, #132777) was used for pegRNA and sgRNA cloning and pU6-tevopreQ1-GG-acceptor (Addgene, #174038) was used for epegRNA cloning. Genotypes of mutants are shown in Table S5. All pegRNAs, nicking sgRNAs and epegRNAs used in this study are provided in the key resources table and Table S6A. PegRNAs designed to install the 77 pathogenic edits into endogenous sites in HEK293T cells were designed using pegRNA spacer and PBS sequences reported previously.^40^ All epegRNA sequences used to install these edits are provided in Table S3.

Fragments assembled after Gibson Assembly were transformed into One Shot Mach1 cells (Thermo Fisher Scientific) and subsequently plated in 2 x YT agar with the appropriate antibiotics. Illustra TempliPhi 100 amplification kit (Cytiva) was used to amplify plasmid DNA before sending it for Sanger sequencing (Quintara Biosciences). Bacterial clones with the verified plasmids were grown in 2 x YT media with the appropriate antibiotics. Plasmid DNA used for mammalian cell transfections were isolated using either QIAGEN Plus Midi Kit or Qiagen Plasmid Plus 96 Miniprep Kit while all other plasmids were isolated using QIAprep Spin Miniprep Kit. All isolated plasmid DNA were eluted in nuclease-free water and quantified using NanoDrop One UV-Vis spectrophotometer (Thermo Fisher Scientific).

#### Phylogenetic tree analysis

RT protein sequences were collected by searching the UniProt database with the BLASTP algorithm using query sequences listed in Table S1. Each individual BLASTP result was filtered to remove duplicate sequences, sequences shorter than 100 residues, and sequences longer than 1000 residues. To reduce phylogenetic complexity, 9–10 representative sequences were randomly sampled from each filtered BLASTP result. The 543 RT sequences used for downstream phylogenetic analyses are listed in Table S1. Phylogenetic analyses were performed using Geneious Prime. The MUSCLE algorithm was used to generate a multiple sequence alignment of all 543 RT sequences. From this sequence alignment, an unrooted tree was generated using the neighbor-joining tree build method with the Jukes-Cantor genetic distance model.

#### Bacteriophage cloning

Phage cloning was performed in a two-step manner as previously described.^62^^,^^63^ Briefly, Gibson Assembly was performed to clone a donor plasmid encoding for either the appropriate reverse transcriptase fused to an Npu C-terminal intein or the entire prime editor protein between two LguI (Life Technologies) type IIS restriction sites. Golden Gate assembly^64^ was performed with the donor plasmid along with two other previously reported plasmids (pBT114-splitC and pBT29-splitD) that each encode for one part of a two-part split phage genome. For Golden Gate assembly, all three plasmids were incubated between 30 min and 18 h with LguI enzyme and T4 DNA ligase at 37°C. Following assembly, the reaction was transformed into chemicompetent S2060^65^ *E. coli* host cells that contain plasmid pJC175e. We refer to this strain as S2208. Plasmid pJC175e supplies gIII under the phage shock promoter, enabling activity-independent phage propagation. After transformation, the cloned phage was grown overnight in Davis Rich Medium (DRM) at 37°C with the appropriate antibiotics. Bacteria were then centrifuged for 5 min at 8,000 g and plaqued (see below). Individual plaques were picked and grown in DRM until the culture reached late growth phase. Bacteria were centrifuged and the supernatant containing phage was isolated. Colony PCR was performed and sent for sanger sequencing (Quintara Biosciences) to confirm that the phage encoded for the correct insert.

#### Preparation of chemically competent cells

Strain S2060 was used in all experiments. Chemically competent cells were prepared as previously described.^66^ Briefly, an overnight culture of bacteria was diluted 50-fold in 2 x YT media with appropriate antibiotics and grown at 37°C, shaking at 230 RPM until the culture reached an optical density (OD_600_) of 0.4–0.6. Cells were then centrifuged at 4°C for 10 min at 4,000g. The supernatant was discarded, and the cell pellets were resuspended in ice-cold TSS solution (LB media supplemented with 5% v/v DMSO, 10% w/v PEG 3350, and 20 mM MgCl_2_). Resuspended cells were aliquoted, frozen in dry ice and stored at −80°C until use.

#### Phage-based luciferase assay

Phage-based luciferase assays were performed as described previously.^63^ For each replicate, one colony of the evolution strain was grown overnight to saturation in DRM and appropriate antibiotics and then back-diluted 50-fold into DRM with appropriate antibiotics. Cultures were grown at 37°C with shaking at 230 RPM until cultures reached OD_600_ = 0.4. The mid-log culture was distributed into a 96-well black clear-bottomed plate (Corning), 135 μL of culture per well. 15 μL of high-titer (1 x10^11^ pfu/mL) phage were added to each well. The plate was covered with a breathable seal and incubated, shaking at 37°C and 230 RPM for 3.5 h. Luminescence and OD_600_ were measured using a plate reader (TECAN). Values reported are OD_600_-normalized luminescence.

#### Plasmid-based luciferase assay

Strains for plasmid-based luciferase assays were made by transforming chemicompetent S2060 *E. coli* with all necessary plasmids, recovering in antibiotic-free DRM for 2 h, and then plating on 2x YT agar containing maintenance antibiotics and 100 mM glucose. For each biological replicate, one colony was picked into DRM and grown overnight. The following day, cultures were back-diluted 50-fold into DRM and antibiotics. For induced samples, arabinose was added to a final concentration of 20 mM. Cultures were grown shaking at 230 RPM and 37°C for 3 h, after which 150 μL were removed, placed into a 96-well black clear-bottomed plate (Corning), and measured for luminescence and OD_600_ on a plate reader (TECAN). Values reported are OD_600_-normalized luminescence.

#### Overnight propagation assay

For each replicate, a single colony of a host strain was picked and grown overnight in DRM and appropriate antibiotics. Saturated cultures were back-diluted 50-fold into DRM with appropriate antibiotics and grown for ∼2 h, at 37°C and 230 RPM until OD reached approximately 0.4. For each phage sample, 1 mL of this mid-log culture was placed into a well of a 96-well deep well plate and then infected with 1E5 total phage. Cultures were grown overnight (37°C and 230 RPM), and then centrifuged for 10 min at 3400g. Supernatant containing phage was collected and then plaqued to determine total number of output phage. Fold propagation is the total number of output phage divided by the number of input phage.

#### Plaquing

Plaquing was performed as previously described.^66^ Briefly, a saturated culture of S2208 *E. coli* was back-diluted 50-fold into DRM containing 50 μg/mL carbenicillin. 2 h later, the mid-log culture (OD = ∼0.5) was used for plaquing. For each phage to be plaqued, three 100-fold serial dilutions of the sample were made using DRM. 10 μL of the original concentrated sample or each serially diluted sample was combined with 100 μL of mid-log 2208 culture. Immediately after mixing the bacteria and the phage, 1 mL of top agar (2:1 ratio of 2x YT media: 2x YT agar, stored at 55°C until use) was added to the phage/bacteria solution, mixed quickly, and then immediately plated on 2x YT agar plates containing no antibiotics and 0.04% Bluogal (Gold Biotechnologies). The following day, the number of blue plaques were counted for whichever dilution (either the concentrated sample or one of the 100-fold dilutions) gave a discernable number of blue plaques. This number was then used to calculate the concentration of the phage sample in pfu/mL. For cases where activity-dependent plaquing was used, the relevant selection strain replaced S2208s.

#### Phage-assisted noncontinuous evolution (PANCE)

To perform one passage of PANCE, chemicompetent selection strains were transformed with MP6,^67^ recovered for 2 h in DRM without antibiotics, and then plated on 2x YT agar plates containing maintenance antibiotics for the selection strain, 25 μg/mL chloramphenicol, and 100 mM glucose. The following day, ∼10 colonies were selected from the plate, pooled in DRM containing 25 μg/mL chloramphenicol and maintenance antibiotics, and grown to OD 0.5. Arabinose was then added to the mid-long culture to reach a final concentration of 20 mM to induce MP6 expression. Immediately after addition of arabinose, 1 mL of this culture per PANCE replicate was infected with 1E5 pfu of phage and then incubated in a 37°C shaker at 230 RPM overnight. The following day, cultures were centrifuged for 10 min at 3400g and the supernatant containing propagating phage was collected and used to infect the next round of evolution. Phage titer after each round was determined using qPCR (see below), Typically, 20 μL of phage were used to infect the next round of evolution (a 1:50 dilution). If phage titers were exceptionally high (1E7 PFU/mL or greater), then a 1:100, 1:200, or 1:1000 dilution factor was used instead. If titers were exceptionally low (less than 1E5 PFU/mL), a passage of drift was performed. For drift passages, 2208s containing MP6 were used instead of selection strains. In drift passages, phage were only allowed to propagate for 6–8 h instead of overnight to minimize recombination-mediated cheating. Once a noticeable change in phage propagation in the selection strain occurred, phage were plaqued using 2208s or the selection strain. Individual plaques were then amplified by PCR using primers JLD 1311 and JLD 1313 (see Table S6B) and submitted for Sanger sequencing to generate inputs for Mutato analysis (https://hub.docker.com/r/araguram/mutato).

#### qPCR determination of PANCE and PACE titers

Phage titers in PANCE were estimated using qPCR as previously described.^66^ For each qPCR titer experiment, in addition to phage pools from evolution, a standard phage sample of a known high titer (1X10^10^ pfu/mL as determined by plaquing) was treated identically to create a standard curve. To titer a phage sample, eight serial 10-fold dilutions of phage were made into DRM (no antibiotics). 25 μL of each serial dilution was heated to 80°C for 30 min. Then 5 μL of heat-treated phage we combined with 44.5 μL of 1x DNase buffer and 0.5 μL of DNase (NEB). The DNase mixture was heated to 37°C for 20 min and then 95°C for 20 min to remove genomes from replication-incompetent polyphage. 1.5 μL of the heat-inactivated DNase mixture was pipetted into a 28 μL Q5 High-fidelity PCR reaction (NEB) containing SYBR Green (Invitrogen) and primers M13-fwd and M13-rev (see Table S6B). qPCR was run on a Biorad CFX96 Real Time system with the following cycling conditions: 98°C for 2 min, [98°C for 10 s, 60°C for 20 s, 72°C for 15 s]x40. Cq values for phage of known titer were used to generate a standard curve, and other samples’ Cq values were used to calculate phage titer in pfu/mL.

#### Phage-assisted continuous evolution (PACE)

Chemicompetent selection strains were transformed with MP6, recovered for 2 h in DRM without antibiotics, and then plated on 2x YT agar plates containing maintenance antibiotics for the selection strain, 25 μg/mL chloramphenicol, and 100 mM glucose. The following day, colonies were picked into DRM and appropriate antibiotics into wells of the top row of a deep well 96-well plate and serially diluted 5-fold down the rows of the plate. The plate was incubated shaking at 37°C and 230 RPM overnight. The next day, wells with an OD_600_ between 0.1 and 0.9 were pooled, diluted to a total volume of 140 mL in DRM and maintenance antibiotics and grown (37°C, 230 RPM) until OD_600_ reached 0.5. This culture was used to fill an 80 mL chemostat and four 15-mL lagoons.

The filled chemostat and lagoons were inserted into a PACE apparatus. Configuration of the PACE apparatus was identical to previously described setups.^66^ The flow rate for the chemostat was controlled by a Masterflex L/S Digital Drive Pump (Cole-Parmer) using a Masterflex L/S Multichannel pump head. Supplement solution for a PACE carboy was made with 500 mL DI water, 59 g Harvard Custom Media C, 50 μL of 0.1M CaCl_2_, 120 μL of a trace metal solution, 400 mg chloramphenicol pre-dissolved in 3 mL of ethanol, and appropriate maintenance antibiotics for the selection strain (500 ng carbenicillin, 1 g spectinomycin, and 300 mg kanamycin, as needed depending on the PACE strain). The supplement was then combined with a 20 L solution of Harvard Custom Media A to create PACE media. This final media was used as input into the chemomstat. The 80 mL chemostat was maintained at OD = ∼0.5, starting with a flow rate of approximately 80 mL/h. The chemostat’s effective flow rate (vol/h) was adjusted throughout the PACE experiment to maintain a constant OD_600_, either by increasing the flow rate on the pump or by decreasing the chemostat volume by lowering the waste needle. Chemostat waste was collected in a carboy containing bleach. Lagoon flow rates were also controlled by a Masterflex L/S Digital Drive Pump (Cole-Parmer) using a Masterflex L/S Multichannel pump head. Mid-log culture from the chemostat was used as the input for all lagoons, and lagoon waste was collected in a carboy containing bleach. To achieve MP6 induction in the lagoons but not the chemostat, arabinose was continuously added to each lagoon. 250 mM arabinose was taken up into a 50 mL syringe, and using a six-channel programmable syringe pump (New Era NE-1600), arabinose was pumped into each lagoon (0.6 mL/h of arabinose for a 15 mL/h lagoon flow rate). The PACE apparatus was allowed to equilibrate for 1–12 h before phage infection.

To begin the PACE, all pumps were turned off, and a total of 1.5E8 pfu were injected into each lagoon. After 10 min, pumps were turned back on, and ∼400 μL was removed from each lagoon for the t = 0 timepoint. Lagoon flow rates began at 0.5 vol/h. Subsequent timepoints were taken every 8–24 h, and each phage sample was stored at 4°C after removal from the lagoon. Immediately after sample collection, lagoon titers were measured using qPCR. If titers were the same as or higher than the previous timepoint, the flow rate was increased by 0.5 vol/h, and arabinose pump rates were adjusted accordingly. If titers were decreasing, flow rate was held constant. Plaquing was used to determine more accurate titers for reporting in figures.

At the end of the PACE experiment, phage were plaqued in two different strains to check for cheating (S2060s to check for gIII recombinants and S2060s transformed with a pT7-gIII plasmid one to check for T7 recombinants), and amplified by PCR to check for bands corresponding to typical cheater recombinants using primers JLD 1311 and JLD 1313. If cheating was not detected (i.e., no plaques on cheater strains and no additional bands via PCR), phage were plaqued in either 2208s or the selection strain. Individual plaques were then amplified by PCR and submitted for Sanger sequencing to generate inputs for Mutato analysis. (https://hub.docker.com/r/araguram/mutato).

#### Transfection of HEK293T, N2a, and Huh7 cells

All transfections used to evaluate editors in mammalian cells were performed in TC-treated 96-well plates (Corning). For both HEK293T cells and N2a cells, a T-75 flask of cells was washed with PBS, trypsinized using TrypLE Express enzyme (Thermo Fisher Scientific), and diluted to a concentration of 1.6E5 cells/mL in DMEM (10% FBS, no antibiotics). 100 μL of diluted cells were added to each well of a 96-well plate. 18–24 h after plating, cells were transfected. For unmodified HEK293T cells, the following conditions were used: 100 ng editor, 40 ng of pegRNA, and 13 ng nicking sgRNA (or, if conducting a twinPE experiment, 40 ng of the other pegRNA) plasmid were combined in a total volume of 6.25 μL Opti-MEM (Thermo Fisher Scientific) per well. For each well, 0.5 μL of Lipofectamine 2000 (Thermo Fisher Scientific) was mixed with 5.75 μL OptiMEM and then combined with the DNA mixture. 10 min later, the DNA/lipid mixture was added dropwise to cells.

For the HEK293T Tay Sachs model cell line, the following conditions were used: 200 ng editor, 40 ng pegRNA, 13 ng nicking sgRNA.

For N2a cells, the procedure was the same as HEK293T cells, except the plasmid DNA amounts differed: for PE3, 175 ng editor, 50 ng pegRNA, and 20 ng nicking sgRNA (or, if conducting a twinPE experiment, 50 ng of the other pegRNA) were used. For PE5 experiments in N2as, 100ng of MLH1dn plasmid was added.

For the twinPE transfection performed in Huh7 cells, 150,000 cells were plated in poly-D-lysine-coated 24-well plates (Corning) in DMEM plus GlutaMAX supplemented with 10% FBS. After 16–24 h, cells were transfected with 400 ng of prime editor plasmid DNA, and 40 ng of each pegRNA plasmid DNA with 2 μL Lipofectamine 2000 (Thermo Fisher Scientific), according to the manufacturer’s protocol.

#### HTS sample preparation

72 h following transfection, cells were washed with PBS (Thermo Fisher Scientific) and lysed for 1 h at 37°C in lysis buffer (10 mM Tris-HCl pH 8, 0.05% SDS and 25 μg/mL proteinase K (Thermo Fisher)). Lysate was then heat inactivated at 80°C for 30 min 1 μL of lysate was used as an input for PCR1. PCR1 reactions were 25 μL total, using the Phusion Hot Start II kit (Thermo Fisher), 0.75 μL of DMSO, and 0.125 μL of each 100μM primer (sequences listed in Table S6B). PCR1 was performed under the following cycle conditions: 98°C for 3 min, [98°C 15 s, 61°C 30 s, 72°C 30 s]x29, 72°C 2 min. Exceptions to these cycling conditions include: N2a sites *Pcsk9* and *Dnmt1* used an annealing temperature of 70°C instead of 61°C, and for twinPE edits, 25 cycles were performed as opposed to 29, in order to decrease PCR bias.

Samples were barcoded in a second PCR reaction (PCR 2). PCR2 reactions were 25 μL total, using the Phusion Hot Start II kit (Thermo Fisher Scientific), 1.25 μL each of 10 μM Illumina barcoding primers, and 1 μL of PCR1. All PCR2 reactions were performed using the following cycling conditions: 98°C for 3 min, [98°C 15 s, 61°C 30 s, 72°C 30 s]x8, 72°C 2 min. After PCR2, samples of similar lengths were pooled and gel extracted in a 1% agarose gel using a Qiaquick gel extraction kit (Qiagen). Concentrations of purified libraries were determined using a Qubit double-stranded DNA high sensitivity kit (Thermo Fisher Scientific) according to the manufacturer’s instructions. Libraries were diluted to 4nM and sequenced using a Miseq (Illumina) using an Illumina Miseq v2 Reagent kit or an Illumina Miseq v2 Micro Reagent kit using single read cycles.

#### HTS analysis

Samples were demultiplexed with Miseq Reporter (Illumina). CRISPResso2 was used to analyze demultiplexed reads. For samples in which the prime edit was a single base change, samples were aligned to the wild type amplicon in batch mode (see Table S6C), using the following parameters: “-q 30”, “-discard_indel_reads TRUE”, and “-qwc”. The value of the qwc parameter, which defined the portion of the sequence to be analyzed for indels, differed for each amplicon. The qwc interval included 10 bp before the first nick of the amplicon (whether that was the prime editing nick site or the PE3 nicking guide nick site) to 10 bp after the second nick of the amplicon (whether that was the prime editing nick site or the PE3 nicking guide nick site). To calculate percent editing, the percent base change was multiplied by an indel correction factor. Percent base changes were found in the CRISPResso2 output file titled “Reference.Nucleotide_percentage_summary.txt”. The indel correction factor was obtained by dividing “reads aligned”/“reads aligned all amplicons” values in the “CRISPResso_quantification_of_editing_frequency.txt” CRISPResso2 output file. To calculate percent indels, “Discarded” was divided by “reads aligned all amplicons” in the same file.

For samples in which the prime edit was multiple base changes or an insertion or deletion, CRISPResso2 was run in HDR batch mode. Parameters were identical to those described above for single nucleotide changes, but an additional parameter “e” was included, the value of which was the sequence of the desired, edited amplicon. For these types of edits, percent editing was calculated by dividing the HDR-aligned reads/reads aligned all amplicons and then multiplying by 100. Indels were calculated by adding the “Discarded” reads from the reference-aligned sequences and the “Discarded” reads from the HDR-aligned sequences and then dividing that sum by “reads aligned all amplicons”. All of these values are found in the “CRISPResso_quantification_of_editing_frequency.txt” file when HDR mode is used.

To quantify scaffold integration, a custom python script available in Note S1 was used. For each condition, scaffold integration is the percentage of (number of amplicons with scaffold-templated bases)/(number of reads that align to the amplicon).

#### *In vitro* transcription (IVT) of editor mRNA

IVT of editor mRNA was performed as described previously.^33^ Editors were cloned into pT7 expression constructs (example Addgene 178113). To generate linear DNA templates for IVT, the pT7-editor plasmids were amplified by PCR using the Phusion U green multiplex master mix (NEB) using primers IVT-fwd and IVT-rev (Table S6B). PCRs were purified using the QIAquick PCR purification kit (Qiagen) and eluted in water. IVT reactions were performed using a T7 high yield RNA synthesis kit (NEB), following the manufacturer’s directions with two exceptions: Trilink’s CleanCap reagent AG was added, and the uridine 5′ triphosphate in the kit was replaced with *N*^1^-methylpseudouridine 5′ triphosphate (Trilink). Each 160 μL reaction used 8 μL 10x reaction buffer, 8 μL 100 mM ATP, 8 μL 100 mM CTP, 8 μL 100 mM GTP, 8 μL 100 mM *N*^1^-methylpseudouridine 5′ triphosphate, 6.4 μL 100 mM CleanCap AG, 16 μL T7 RNAP mix, and 1 μg of purified linear template DNA. After assembly, reactions were incubated at 37°C for 4 h. Samples were then DNase treated by adding 544 μL water, 80 μL DNase reaction buffer (NEB), and 60 μL DNaseI (NEB) to the IVT reaction. Samples were incubated at 37°C for 15 min, and RNA was purified using a lithium chloride precipitation, following by two washes in 70% ethanol. RNA was resuspended in nuclease-free water, and purity and quality were verified using a 2% agarose gel stained with SYBER Gold (Thermo Fisher Scientific). RNA was stored at −80 until use.

#### Electroporation of patient-derived fibroblasts

An 80% confluent T-75 flask of patient-derived fibroblasts (Coriell) were washed with PBS (Thermo Fisher Scientific), trypsinized using TrypLE Express enzyme (Thermo Fisher Scientific), and suspended in 10 mL of media. The following media was used for each patient-derived fibroblast line: low-glucose DMEM (Sigma Aldrich) supplemented with 10% (v/v) FBS and 2mM GlutaMAX Supplement (Thermo Fisher Scientific) for Tay Sachs Disease (ID: GM00221), high-glucose DMEM (Thermo Fisher Scientific) supplemented with 15% (v/v) FBS and 2mM GlutaMAX Supplement (Thermo Fisher Scientific) for Pompe Disease (ID: GM20092) and EMEM (ATCC) supplemented with 15% (v/v) FBS for both Crigler-Najjar Syndrome (ID: GM09551) and Bloom Syndrome (ID: GM02085). Cells were transferred to falcon tubes and centrifuged for 5 min at 150 g. During centrifugation, RNA reagents were prepared. For each sample, 1 μL of 1 μg/μL editor mRNA was added to a PCR tube, along with 0.45 μL of a 200 μM *HEXA*1278ins correction pegRNA solution and 0.6 μL of a 100 μM *HEXA*1278ins correction nicking sgRNA solution. (See key resources table for sequences of epegRNA and nicking sgRNA). An SE cell line kit (Lonza) was used to perform electroporation. 90.2 μL of SE nucleofector solution was mixed with 19.8 μL of supplement solution to make reconstituted Lonza buffer. Pelleted cells were washed with PBS and resuspended in the reconstituted Lonza buffer. 20 μL of resuspended cells was added to each editor/epegRNA/nicking guide mixture, transferred to a cuvette (Lonza), and electroporated using program CM130 on a Lonza 4D nucleofector with X unit (100,000 cells per electroporation condition). Immediately after electroporation, 80 μL of media was added to each well and incubated at room temperature for 10 min 1 mL of media was aliquoted into each well of a 24 well plate, and all cells were transferred to this plate. Cells grew for 5 days, with a media change at day 3, before lysis and sequencing.

#### Electroporation of primary human T cells

T cells were cultured in X-VIVO TM 15 Serum-free Hematopoietic Cell Medium (Lonza, Basel, Switzerland) supplemented with: 300 IU/mL IL-2 (PeproTech, Cranbury, NJ), GlutaMAX (Gibco, Waltham, MA), N-acetyl-cysteine (Sigma Aldrich, St. Louis, MO), 5% AB human serum (Valley Biomedical, Winchester, VA), 50 U/mL penicillin and 50 μg/mL streptomycin (Gibco, Waltham, MA). T-cells were stimulated with a 3:1 ratio of Dynabeads Human T-Expander CD3/CD28 beads (Thermo Fisher Scientific, Waltham, MA) and cells. At 72 h, the beads were removed and 300,000 T-cells were electroporated with 1 μL (1 μg) of editor mRNA, 1 μL (2 μg) of MLH1dn mRNA, 0.9 μL (100 μM) pegRNA, and 0.6 μL (100 μM) nicking sgRNA using the Neon electroporation system (ThermoFisher) with 10 μL tips and instrument settings of 1,400 V, 10 ms, and 3 pulses. Cells were cultured for 72 h followed by DNA isolation using the QuickExtract DNA Extraction Solution.

#### TDT assay and analysis

HEK293T cells were transfected in a 96 well plate as described above using 200 ng of editor and 40 ng of pegRNA. (No nicking guides were used for TDT transfections). 24 h after transfection, cells were lysed using 50 μL of lysis buffer per well (47.5 μL Beckman lysis Buffer (Beckman Coulter), 1.25 μL of 1M DTT, and 1.25 μL of proteinase K (Thermo Fisher). Genomic DNA was purified using the Beckman bead purification kit (Beckman Coulter) and eluted in 40 μL of water. 10 μL of purified genomic DNA was used in a 50 μL tailing reaction (1X TDT buffer, 0.25 mM CoCl_2_, 100 μM dGTP, 10 units of terminal transferase, NEB). Samples were incubated at 37°C for 30 min and then 70°C for 10 min. The tailed DNA was isolated from the reaction mixture using the Beckman bead purification kit again and eluted in 20 μL of water. 5 μL of purified tailed DNA was used as input for a 50 μL PCR1 reaction. TDT PCR1 reactions were performed with Phusion U Green Multiplex PCR Master Mix (25 μL), 5 μL of purified tailed DNA, 19.5 μL of water, and 0.25 μL of 100 μM primers. For TDT assay sequencing, one site-specific primer and one polyC primer (see Table S6B) were used for PCR1. PCR2 and Miseq were then performed as described above in “HTS sample preparation”.

To analyze TDT samples, a custom Python script (Note S2) was used to analyze demultiplexed fastq files. For scaffold insertion plots (Figure S4F), TDT results are plotted as the percentage of total edit-containing flaps of a given length. For plots showing the lengths of RTT-encoded flaps synthesized (Figures 4D and S4C), all RT products (flaps length 1 or more) were counted, regardless of whether or not they contained the entire edit. Because polyG tailing was used, flap lengths corresponding to a flap ending in G are not detected.

#### Secondary structure preduction using NUPACK^38^

Using the “old” NUPACK website (https://old.nupack.org/), the sequence of the pegRNA RTT and PBS was entered as the strand1 sequence using the RNA setting, a temperature of 37°C, and default other parameters. This measure of folding free energy does not consider the pegRNA spacer, scaffold, or epegRNA 3′ pseudoknot motif, as they are not directly engaged by the RT.

#### UMI sample prep and analysis

Unique molecular identifiers (UMIs) were applied in a three-step PCR protocol as previously described.^9^ Briefly, linear amplification was first performed with 1uL of genomic DNA, Phusion U Green Multiplex PCR Master Mix and 0.1 μM of only the forward primer containing a 15-nt UMI in a 25 μL reaction (eleven cycles of 98°C for 1 min, 61°C for 25 s and 72°C for 1 min). 1.6x AMPure beads (Beckman Coulter) was used to purify the PCR products in 20 μL nuclease-free water, according to the manufacturer’s protocol. For the second PCR, a forward primer that binds to the P5 Illumina adaptor sequence located at the 5′ end of the UMI primer was used. This PCR was performed using 2uL of purified linear DNA, 0.5 μM of each forward and reverse primer and Phusion U Green Multiplex PCR Master Mix for 30 cycles in a 25 μL reaction. In the third PCR, 1 μL of product from the second PCR was amplified for 10 cycles using Phusion U Green Multiplex PCR Master Mix to add unique Illumina barcodes and adaptors as has been described earlier. The products from the third PCR were then pooled, separated by electrophoresis on a 1% agarose gel and purified with QIAquick Gel Extraction Kit (QIAGEN). The library was quantified using Qubit 3.0 Fluorometer (Thermo Fisher Scientific) and finally sequenced using the MiSeq Reagent Kit v2 or MiSeq Reagent Micro Kit v2 (Illumina) with 300 single-read cycles. AmpUMI^60^ was used to UMI deduplicate the raw sequencing reads. The UMI-deduplicated R1s were then analyzed using CRISPResso2 as described earlier.^59^

#### AAV production

Transfer vectors were designed and generated as previously described (see v3em constructs from Davis et al.^25^). epegRNA sequences were changed to change the target edit. For transfer vectors using PE6c, further truncation of the Tf1 RT allowed us to minimize prime editor size an additional 100 bp to facilitate AAV packaging. For the single flap *loxP* insertion single flap edit at the *Dnmt1* locus, the 40-bp *loxP* sequence was inserted, along with 2 additional bp of filler sequence to preserve the frame of the *Dnmt1* open reading frame after editing.

AAV production was performed as previously described.^25^^,^^68^ HEK293T/17 cells (ATCC) were cultured in DMEM with 10% fetal bovine serum without antibiotics in 150-mm^2^ dishes (Thermo Fisher Scientific) and passaged every 2–3 days at 37°C with 5% CO_2_. Cells were split 1:3, 18–22 h before transfection. 5.7 μg AAV genome, 11.4 μg pHelper (Clontech), and 22.8 μg AAV9 rep-cap plasmid were transfected per plate using polyethyleneimine (PEI MAX, Polysciences). Media was exchanged for DMEM with 5% fetal bovine serum the following day. Three days after the media change, cells were harvested using a rubber cell scraper (Corning), pelleted via centrifugation (10 min, 2,000 *g*) and resuspended in 500 μL hypertonic lysis buffer (40 mM Tris base, 2 mM MgCl_2_, 500 mM NaCl, and 100 U mL^−1^ salt active nuclease (ArcticZymes)) per plate, and incubated at 37°C for 1 h. The media was decanted and combined with 5x solution of poly(ethylene glycol) (PEG) 8000 (Sigma-Aldrich) and NaCl to achieve a final concentration of 8% PEG and 500 mM NaCl. This solution was incubated on ice for 2 h or overnight to facilitate PEG precipitation and then centrifuged (3,200 *g*, 30 min). The supernatant was discarded, and the pellet was resuspended in 500 μL hypertonic lysis buffer per plate. This was added to the cell lysate, which was either immediately ultracentrifuged or stored at 4°C overnight.

Cell lysates were first clarified by centrifugation at 3,400 *g* for 10 min and added to Beckman Coulter Quick-Seal tubes using a 16-gauge, 5-inch needle (Air-Tite N165) in a discontinuous gradient of iodixanol. The gradient of iodixanol was formed by sequentially floating the following layers: 9 mL 15% iodixanol in 500 mM NaCl and 1x PBS-MK (1x PBS with 2.5 mM KCl, and 1 mM MgCl_2_), 6 mL 25% iodixanol in 1x PBS-MK, and 5 mL each of 40% and 60% iodixanol in 1x PBS-MK. Phenol red was added to a final concentration of 1 μg mL^−1^ in the 15, 25, and 60% layers to facilitate layer identification. Ultracentrifugation was performed at 58,600 rpm for 2 h 15 min at 18°C using a Ti 70 rotor in an Optima XPN-100 Ultracentrifuge (Beckman Coulter). After centrifugation, an 18-gauge needle was used to remove 3 mL of solution from the 40–60% iodixanol interface. This solution was buffer exchanged using PES 100 kD MWCO columns (Thermo Fisher Scientific) with cold PBS containing 0.001% F-68 and finally sterile filtered using a 0.22-μm filter. The final concentrated AAV solution was quantified using qPCR (AAVpro titration kit, Clontech) and stored at 4°C until use.

#### Animals

All mouse experiments were approved by the Broad Institute Institutional Animal Care and Use Committee and consistent with local, state, and federal regulations (as applicable), including the National Institutes of Health Guide for the Care and Use of Laboratory Animals. For P0 studies, timed pregnant C57BL/6J mice were purchased from Charles River Laboratory. All mice were housed in a room maintained on a 12 h light and dark cycle with *ad libitum* access to standard rodent diet and water.

#### P0 ventricle injections

All *in vivo* editing experiments were conducted via an ICV injection performed on day P0. P0 ventricle injections were performed as described previously.^25^^,^^68^ Drummond PCR pipettes (5-000-1001-X10) were pulled at the ramp test value of a Sutter P1000 micropipette puller and passed through a Kimwipe three times to achieve a tip diameter size of ∼100 μm. To assess ventricle targeting, a small amount of Fast Green dye was added to the AAV injection solution. Using the included Drummond plungers, 4 μL of the injection solution was loaded via front filling. Cryoanestheisa was used to anesthetize the P0 pups. Successful anesthesia was verified by color and unresponsiveness to bilateral toe pinch. Then, 2μL of the injection solution was injected freehand into each ventricle. Transillumination of the head was used to assess ventricle targeting by the spread of Fast Green throughout the ventricles. Genders of mice and viral doses used for *in vivo* experiments are as follows (M = male, F = female, vg = viral genomes):

Low-dose twinPE attB ins: [PEmaxΔRNaseH: 3M + 1F, PE6d: 2M +2F, PE6c: 2M + 2F, untreated 3F]. Treated mice received 2E10 vg of each PE virus and 1E10 vg of GFP-KASH virus.

Low-dose PE *loxP* ins. [PEmaxΔRNaseH: 2M + 1F, PE6d: 2M + 1F, untreated: 1M]. Treated mice received 1E10 vg of each PE virus and 1E10 vg of GFP-KASH virus.

High-dose PE *loxP* ins. [PEmaxΔRNaseH: 3M, PE6d: 2M + 1F, untreated 1M, 2F]. Treated mice received 5E10 vg of each PE virus and 1E10 vg of GFP-KASH virus.

We note that the prime editor AAV doses used in these experiments (1.35x10^13^ total vg/kg to 6.75x10^13^ total vg/kg) is 1.6-fold–8-fold lower than the 1.1x10^14^ vg/kg dose used in FDA-approved AAV therapies.^69^

#### Mice tissue collection

All mice were sacrificed by CO_2_ asphyxiation, and tissues were immediately dissected. To harvest the cortex, hemispheres were first split sagittally using a razor blade. The cortex (neocortex + hippocampus) was then isolated using a microspatula.

#### Nuclear isolation and sorting

Nuclear isolation and sorting were performed as described previously.^25^^,^^68^ Dissected cortex tissue was first homogenized using a glass Dounce homogenizer (Sigma-Aldrich; D8938) with 20 strokes of pestle A followed by 20 strokes of pestle B in 2 mL of ice-cold EZ-PREP buffer ((Sigma-Aldrich). Sample was decanted into a new tube with additional 2 mL of cold EZ-PREP buffer on ice and centrifuged (500*g,* 4°C). The supernatant was decanted, and the nuclei pellet was resuspended in 4 mL of ice-cold Nuclei Suspension Buffer (NSB: 100 mg/mL BSA (New England Biolabs) and 3.33 mM Vybrant DyeCycle Ruby (Thermo Fisher) in PBS). The sample was again centrifuged at 500*g* for 5 min at 4°C, the supernatant was decanted, and the nuclei were resuspended in 1 mL of NSB. Samples were passed twice through a 35-μM cell strainer before flow sorting using the Sony MA900 Cell Sorter (Sony Biotechnology) at the Broad Institute flow cytometry core. See Figure S7B for example FACS gating. Nuclei were sorted into DNAdvance lysis buffer, and the genomic DNA was purified according to the manufacturer’s protocol (Beckman Coulter).

#### Analysis of off-target editing

Previously identified murine *Dnmt1* off-target sites^26^^,^^50^ were amplified from either bulk or sorted cells from the mouse cortex. One of the off-target sites did not amplify efficiently by PCR. CRISPRESSO was run without an e flag (not in HDR mode), with indels discarded, and with a w value of 20. Off-target edits were counted as leniently as possible: percent off-targets was calculated as the sum of indel reads and editing reads divided by the total number of reads aligned for all amplicons x 100. Off-target indels were counted as the number of discarded reads for the sample. To calculate off-target editing events, the pegRNA-encoded sequence was compared to the off-target site. The first SNP at which the two sequences differed was used as a marker for off-target editing: all reads containing that SNP were counted as off-target editing events, even if they did not contain the entire *loxP* insertion.

### Quantification and statistical analysis

The number of independent biological replicates and technical replicates for each experiment are described in the figure legends or the STAR Methods section.

## Data Availability

• All sequencing data have been deposited at the NCBI Sequence Read Archive database and are publicly available as of the date of publication. Accession numbers are listed in the key resources table. • All original code is available in Notes S1 and S2. • Any additional information required to reanalyze the data reported in this paper is available from the lead contact upon request.

## Supplemental information

Document S1. Notes S1 and S2

Table S1. List of reverse transcriptases used in this study, related to Figure 1 Sequences and identities of reverse transcriptases used for BLAST query, phylogenetic analysis, and initial screen of RTs in Figures 1B and 1C

Table S2. (A–N) Mutation tables from PE PA(N)CE, related to Figures 2, 3, 4, and 6 Mutations in clones emerging from evolution

Table S3. Reagents used to install pathogenic mutations into endogenous sites in HEK293T cells using PEmax and Tf1-derived editors along with their corresponding editing and indel frequencies, related to Figure 5 EpegRNAs, nicking sgRNAs, high-throughput sequencing primers, and amplicons, along with editing and indel frequencies used to make Figure 5F

Table S4. Editing efficiencies of single Cas9 mutants, related to Figure 6 Summary of mutants and corresponding editing efficiencies used to generate Figure 6E

Table S5. Genotypes of RT and Cas9 variants related to Figures 1, 3, 4, 5, and 6 Mutations in RT and Cas9 variants that were tested in Figures 1, 3, 4, 5, and 6

Table S6. (A–C) pegRNA sequences, primer sequences, and amplicon sequences for HTS analysis used in this study, related to Figures 1–7
