# Supplementary material for: Phage-assisted evolution and protein engineering yield compact, efficient prime editors
Source: Cell. 2023 Aug 31;186(18):3983–4002.e26. doi: 10.1016/j.cell.2023.07.039 (PMC10482982; doi:10.1016/j.cell.2023.07.039)
Supplement: Document S1. Notes S1 and S2 [file mmc1.pdf]

Cell, Volume *186*

Supplemental information

Phage-assisted evolution and protein engineering yield compact, efficient prime editors

Jordan L. Doman, Smriti Pandey, Monica E. Neugebauer, Meirui An, Jessie R. Davis, Peyton B. Randolph, Amber McElroy, Xin D. Gao, Aditya Raguram, Michelle F. Richter, Kelcee A. Everette, Samagya Banskota, Kathryn Tian, Y. Allen Tao, Jakub Tolar, Mark J. Osborn, and David R. Liu

Supplementary Notes

Phage-assisted evolution and protein engineering yield compact, efficient prime editors

Jordan L. Doman[1,2,3,5], Smriti Pandey[1,2,3,5], Monica E. Neugebauer[1,2,3], Meirui An[1,2,3], Jessie R. Davis[1,2,3], Peyton B. Randolph[1,2,3], Amber McElroy[4], Xin D. Gao[1,2,3], Aditya Raguram[1,2,3], Michelle F. Richter[1,2,3], Kelcee A. Everette[1,2,3], Samagya Banskota[1,2,3], Kathryn Tian[1,2,3], Y. Allen Tao[1,2,3], Jakub Tolar[4], Mark J. Osborn[4], David R. Liu[1,2,3,*]

[1] Merkin Institute of Transformative Technologies in Healthcare, Broad Institute of MIT and Harvard, Cambridge, MA, USA.
[2] Department of Chemistry and Chemical Biology, Harvard University, Cambridge, MA, USA.
[3] Howard Hughes Medical Institute, Harvard University, Cambridge, MA, USA.
[4] Department of Pediatrics, University of Minnesota Medical School, Minneapolis, MN, USA
[5] These authors contributed equally

*Lead Contact: drliu@fas.harvard.edu

Supplementary Note 1. Custom python script used for quantification of pegRNA insertions at the target genomic locus (First reported in Anzalone et al., 2019), related to STAR methods.

```
## sgRNA scaffold sequence search ##

import pandas as pd
import Bio as bio
from Bio import SeqIO
import glob

#generates list of fastq files to analyze
sources = glob.glob('*.fastq')

#reads the fastq files into a dictionary with the file names as keys
fastqdict = {}
for i in range(len(sources)):
	temp = list(SeqIO.parse(sources[i],"fastq"))
	fastqdict[sources[i]]= [str(temp[k].seq) for k in range(len(temp))]

#the referenced sequence to be searched for is entered into the following dictionary with
#an appropriate key

scaffdict =
{'RNF2':CTGATGTGTTCGTTGCACCGACTCGGTGCCACTTTTTCAAGTTGATAACGGACTAGCCTT
ATTTTAACTTGCTATTTCTAGCTCTAAAACCAGGTAATGACTAAGATGAC',
'PRNP':'TGGCGTCTACATGCACCGACTCGGTGCCACTTTTTCAAGTTGATAACGGACTAGCCTTAT
TTTAACTTGCTATTTCTAGCTCTAAAACCCAAGGCCCCCCACCACTGC',
'FANCF':'ACCTTGATCGCTTTTCCGCACCGACTCGGTGCCACTTTTTCAAGTTGATAACGGACTAG
CCTTATTTTAACTTGCTATTTCTAGCTCTAAAACGGTGCTGCAGAAGGGATTCC',
'EMX1':'GAAGTGCTCCCATCACGCACCGACTCGGTGCCACTTTTTCAAGTTGATAACGGACTAGC
CTTATTTTAACTTGCTATTTCTAGCTCTAAAACTTCTTCTTCTGCTCGGACTC',
'VEGFA':'TGAGTGCTCCAGATGGCACATTGCACCGACTCGGTGCCACTTTTTCAAGTTGATAACG
GACTAGCCTTATTTTAACTTGCTATTTCTAGCTCTAAAACTCATCTGGCCTGCAGACATC',
'Ctnnb1':'TCAGGAAAGGAGCGCACCGACTCGGTGCCACTTTTTCAAGTTGATAACGGACTAGCCTT
ATTTTAACTTGCTATTTCTAGCTCTAAAACTGAGTGGCAAGGGCAACCCTC',
}

#matches and counts iterative slices of the reference string to the appropriate fastq files #reference
key must be contained in the name of the fastq file
#generated values represent cumulative counts for a minimum degree of sgRNA integration
#i.e. a given value x means x reads contain y or more bases of the scaffold

resultdict = dict.fromkeys(sources)
for key in fastqdict:
	for scaffold in scaffdict:
		if scaffold in str(key):
			resultlist = []
			for j in range(len(scaffdict[scaffold])):
				extent = scaffdict[scaffold][0:(j+1)]
				counter = 0
				for i in range(len(fastqdict[key])):
					if extent in fastqdict[key][i]:
						counter = counter + 1
```

```
                resultlist.append(counter)
            resultdict[key]=resultlist

#writes the results into a dataframe indexed from 1
resultdf = pd.DataFrame.from_dict(resultdict)
resultdf = resultdf.reindex(sorted(resultdf.columns), axis=1)
resultdf.index = range(1,len(resultdf)+1)

#converts the cumulative count values into specific counts
#i.e. a given value x means x reads contain exactly y bases of the scaffold resultdf2=resultdf.copy()
for entry in resultdf:
    for i in range(1,len(resultdf[entry])+1):
        try:
            resultdf2[entry][i] = resultdf[entry][i]-resultdf[entry][i+1]
        except:
            resultdf2[entry][i] = resultdf[entry][i]

#converts the specific counts values into frequencies
resultdf3=resultdf2.copy()
for entry in resultdf3:
    resultdf3[entry]=resultdf2[entry].div(resultdf[entry][1])*100

#reads the results into excel files
resultdf.to_excel('cumulativecounts.xlsx')
resultdf2.to_excel('specificcounts.xlsx')
resultdf3.to_excel('specificfrequencies.xlsx')
```

Supplementary Note 2. Custom python script used for analyzing TDT sequencing. (Modified from Nelson et al. 2021), related to STAR methods.

```
import pandas as pd
import glob
import re
import os
import subprocess
from subprocess import Popen
from subprocess import PIPE
import Bio as bio
from Bio import SeqIO
from Bio.Seq import Seq
import collections

#if analyzing files where the spacer occurs in the reverse complement of the FASTQ reads, set
#revcomp_mode to True

revcomp_mode=False

fastqs = glob.glob('*.fastq')
first10nts = {
             'HEK3':'GGCCCAGACT',
             'DNMT1':'GATTCCTGGT',
            'RNF2':'GTCATCTTAG',
             'RUNX1':'GCATTTTCAG',
             'VEGFA':'GATGTCTGCA',
            'FANCF':'GGAATCCCTT',
            'EMX1':'GAGTCCGAGC',
           'CCR5':'GTATGGAAAA'

            }

if revcomp_mode:
    for fname in fastqs:
        fastqs_rev = list(str(k.seq.reverse_complement()) for k in SeqIO.parse(fname,"fastq"))
        with open(f'{fname[:-6]}_trimmed.txt','w+') as f:
                for spacer in first10nts.keys():
                    if spacer in fname:
                        for entry in fastqs_rev:
                            nt_read_re = re.search(first10nts[spacer]+'(.*?)GGGGGGGG',entry)
                            try: f.write(str(nt_read_re.group(0))+'\n')
                            except: continue
else:
    for fname in fastqs:
        with open(f'{fname[:-6]}_trimmed.txt','w+') as f:
            for spacer in first10nts.keys():
                if spacer in fname:

                    nt_readARGS = ['grep', '-o', f'{first10nts[spacer]}.*GGGGGGGG', fname]
                    nt_readproc = Popen(nt_readARGS, stdout=subprocess.PIPE, universal_newlines=True)
                    f.write(str(nt_readproc.stdout.read())+'\n')
```

```
#Include reverse complement of spacer in scaffold_revcomp
#If analyzing RT products encoding insertions, set insert_len to length of the insert and edit_pos to
#where the first mismatched base occurs
#If running in frequency batch mode, set frequency_batch to True

frequency_batch = True

if frequency_batch:
    amplicondict=collections.defaultdict(list)

trimmedfastqs = glob.glob('*trimmed.txt')

for fname in trimmedfastqs:
    sequences_b = open(fname, 'r')

    if 'HEK3' in fname:
        amplicon='HEK3'
        edit_pos = 1
        designed_flap =
'GATTACAAGGATGACGACGATAAGTGATGGCAGAGGAAAGGAAGCCCTGCTTCCTCCA'
        RT_temp_length = 58
        insert_len = 24
        scaffold_revcomp =
'GCACCGACTCGGTGCCACTTTTTCAAGTTGATAACGGACTAGCCTTATTTTAACTTGCTATTTCTA
GCTCTAAAACTCACGTGCTCAGTCTGGGCC'
    if 'RNF2' in fname:
        amplicon='RNF2'
        edit_pos = 5
        designed_flap = 'CTGATGTGTTCGTT'
        RT_temp_length = 14
        insert_len = 0
        scaffold_revcomp =
'GCACCGACTCGGTGCCACTTTTTCAAGTTGATAACGGACTAGCCTTATTTTAACTTGCTATTTCTA
GCTCTAAAACCAGGTAATGACTAAGATGAC'
    if 'VEGFA' in fname:
        amplicon='VEGFA'
        edit_pos = 1
        designed_flap = 'GATTACAAGGATGACGACGATAAGTGAGTGCTCCAGATGGCACATT'
        RT_temp_length = 46
        scaffold_revcomp =
'GCACCGACTCGGTGCCACTTTTTCAAGTTGATAACGGACTAGCCTTATTTTAACTTGCTATTTCTA
GCTCTAAAACTCATCTGGCCTGCAGACATC'
    if 'DNMT1' in fname:
        amplicon='DNMT1'
        edit_pos = 5
        designed_flap = 'ACAGTGGTGAC'
        RT_temp_length = 11
        insert_len = 0
        scaffold_revcomp =
'GCACCGACTCGGTGCCACTTTTTCAAGTTGATAACGGACTAGCCTTATTTTAACTTGCTATTTCTA
GCTCTAAAACGCGCGAACAGCTCCAGCCCGC'
    if 'FANCF' in fname:
        amplicon='FANCF'
```

```
        edit_pos = 5
        designed_flap = 'ACCTTGATCGCTTTTCC'
        RT_temp_length = 17
        scaffold_revcomp =
'GCACCGACTCGGTGCCACTTTTTCAAGTTGATAACGGACTAGCCTTATTTTAACTTGCTATTTCTA
GCTCTAAAACGGTGCTGCAGAAGGGATTCC'
    if 'EMX1' in fname:
        amplicon='EMX1'
        edit_pos = 5
        designed_flap = 'GAAGTGCTCCCATCAC'
        RT_temp_length = 16
        insert_len = 0
        scaffold_revcomp =
'GCACCGACTCGGTGCCACTTTTTCAAGTTGATAACGGACTAGCCTTATTTTAACTTGCTATTTCTA
GCTCTAAAACTTCTTCTTCTGCTCGGACTC'


    seq_list = []

    df = pd.DataFrame({'flap seq' : [],'flap length' : [],'contains edit?' : [],'scaffold insertion length' : []})

    for line in sequences_b:
        seq = str(line)
        seq_list.append(seq)

    counter = 0
    counter_b = 1
    for read in seq_list:
        edit = 0
        scaff_RT = 0
        for k in range(len(read) - 5):
            window = read[k:k+5]
            if window == 'GGGGG':
                spacer_flap = read[0:k]
                flap_length = len(spacer_flap) - 17
                if flap_length > edit_pos:
                    if flap_length < len(designed_flap):
                        three_prime = flap_length
                    else:
                        three_prime = len(designed_flap)
                    if insert_len > 0:
                        if len(spacer_flap[17:17+three_prime]) >= (insert_len+edit_pos-1):
                            if spacer_flap[17:17+three_prime] == designed_flap[:three_prime]:
                                edit = 1
                    elif spacer_flap[17:17+three_prime] == designed_flap[:three_prime]:
                        edit = 1
                if flap_length > RT_temp_length:
                    scaff_ins_len = flap_length - RT_temp_length
                    scaff_ins_seq = spacer_flap[RT_temp_length+17:]
                    if scaffold_revcomp[0:scaff_ins_len] == scaff_ins_seq:
                        scaff_RT = scaff_ins_len
```

```
                new_row = pd.DataFrame({'flap seq' : [spacer_flap],'flap length' : [flap_length],'contains
edit?' : [edit],'scaffold insertion length' : [scaff_RT]})
                df=pd.concat([df,new_row], ignore_index=True)
                df.reset_index()
                break

    df = df[['flap seq','flap length','contains edit?','scaffold insertion length']]

    df.to_csv(f'{fname[:-4]}_output.csv', index=False)

    if frequency_batch:
        filedict={}
        flapdict={}
        for i in range(len(df)):
            if df.iloc[i,2]==1.0:
                flapdict[df.iloc[i,0]]=flapdict.get(df.iloc[i,0],0)+1
        flap_denom = sum(flapdict.values())
        for k,v in flapdict.items():
            flapdict[k]=v/flap_denom
        filedict[fname]=flapdict
        filedf = pd.DataFrame(filedict)
        amplicondict[amplicon].append(filedf)

for amplicon in amplicondict.keys():
    amplicondf=pd.concat(amplicondict[amplicon],axis=1)
    amplicondf['flap length'] = [len(i) for i in amplicondf.index]
    amplicondf.sort_values(['flap length'], ascending=True, inplace=True)
    amplicondf.to_csv(f'{amplicon}_flapfrequencies.csv')
```
